# Exploring the Relationship Among Divergence Time and Coding and Non-coding Elements in the Shaping of Fungal Mitochondrial Genomes

**DOI:** 10.3389/fmicb.2020.00765

**Published:** 2020-04-29

**Authors:** Paula L. C. Fonseca, Fernanda Badotti, Ruth B. De-Paula, Daniel S. Araújo, Dener E. Bortolini, Luiz-Eduardo Del-Bem, Vasco A. Azevedo, Bertram Brenig, Eric R. G. R. Aguiar, Aristóteles Góes-Neto

**Affiliations:** ^1^Molecular and Computational Biology of Fungi Laboratory, Department of Microbiology, Instituto de Ciências Biológicas, Universidade Federal de Minas Gerais, Belo Horizonte, Brazil; ^2^Department of Chemistry, Centro Federal de Educação Tecnológica de Minas Gerais, Belo Horizonte, Brazil; ^3^Department of Molecular and Cellular Oncology, The University of Texas MD Anderson Cancer Center, Houston, TX, United States; ^4^Program of Bioinformatics, Instituto de Ciências Biológicas, Universidade Federal de Minas Gerais, Belo Horizonte, Brazil; ^5^Department of Botany, Instituto de Ciências Biológicas, Universidade Federal de Minas Gerais, Belo Horizonte, Brazil; ^6^Institute of Veterinary Medicine, Burckhardtweg, University of Göttingen, Göttingen, Germany

**Keywords:** mitogenomes, Ascomycota, non-coding elements, structural variation, gene transfer, divergence time

## Abstract

The order Hypocreales (Ascomycota) is composed of ubiquitous and ecologically diverse fungi such as saprobes, biotrophs, and pathogens. Despite their phylogenetic relationship, these species exhibit high variability in biomolecules production, lifestyle, and fitness. The mitochondria play an important role in the fungal biology, providing energy to the cells and regulating diverse processes, such as immune response. In spite of its importance, the mechanisms that shape fungal mitogenomes are still poorly understood. Herein, we investigated the variability and evolution of mitogenomes and its relationship with the divergence time using the order Hypocreales as a study model. We sequenced and annotated for the first time *Trichoderma harzianum* mitochondrial genome (mtDNA), which was compared to other 34 mtDNAs species that were publicly available. Comparative analysis revealed a substantial structural and size variation on non-coding mtDNA regions, despite the conservation of copy number, length, and structure of protein-coding elements. Interestingly, we observed a highly significant correlation between mitogenome length, and the number and size of non-coding sequences in mitochondrial genome. Among the non-coding elements, group I and II introns and homing endonucleases genes (HEGs) were the main contributors to discrepancies in mitogenomes structure and length. Several intronic sequences displayed sequence similarity among species, and some of them are conserved even at gene position, and were present in the majority of mitogenomes, indicating its origin in a common ancestor. On the other hand, we also identified species-specific introns that advocate for the origin by different mechanisms. Investigation of mitochondrial gene transfer to the nuclear genome revealed that nuclear copies of the *nad5* are the most frequent while *atp8*, *atp9*, and *cox3* could not be identified in any of the nuclear genomes analyzed. Moreover, we also estimated the divergence time of each species and investigated its relationship with coding and non-coding elements as well as with the length of mitogenomes. Altogether, our results demonstrated that introns and HEGs are key elements on mitogenome shaping and its presence on fast-evolving mtDNAs could be mostly explained by its divergence time, although the intron sharing profile suggests the involvement of other mechanisms on the mitochondrial genome evolution, such as horizontal transference.

## Introduction

The mitochondrion is an important organelle of eukaryotic cells, involved in multiple processes that are necessary for cell survival and reproduction. It is responsible for the ATP production, the universal energy-transfer biomolecule in living organisms, through the oxidative phosphorylation pathway. In addition to respiratory metabolism and energy production function, mitochondria is also involved in other processes such as senescence during the cell cycle and the maintenance of ion homeostasis (Burger et al., 2003; Basse, 2010; Chatre and Ricchetti, 2013).

Conversely to the majority of other intracellular membrane structures, mitochondria contain their own genome that is capable of independent replication and inheritance. Nonetheless, over evolutionary time, most of the genes in the initial endosymbiont have been transferred to the nuclear genome and only a few genes are retained in mitochondrial genomes (mitogenomes). Indeed, most of the mitochondrial proteins are encoded by the nuclear genome and transported to the mitochondria (Burger et al., 2003). Within each mitochondrion, there are typically multiple copies of the genome, which replicate independently of the nuclear genome and cell cycle (Chatre and Ricchetti, 2013). Furthermore, most of the mitochondrial genomes are composed of a single circular chromosome, although some organisms contain linear mitochondrial genomes made up of multiple subunits (Burger et al., 2003).

Fungal mitogenomes are generally characterized by the presence of 14 conserved protein-coding genes involved in electron transport (*cytochrome oxidase subunits 1*, *2*, and *3*, *NADH dehydrogenase subunits 1*, *2*, *3*, *4*, *4L*, *5*, and *6*) and ATP synthesis (*ATP synthase subunits 6*, *8*, and *9*, and *apocytochrome b*). In addition, mitochondrial genome typically has one of the small (SSU rRNA) and large subunits ribosomal RNAs (LSU rRNA), and a set of transfer RNA (tRNA) genes (Bullerwell and Lang, 2005; Al-Reedy et al., 2012; Ferandon et al., 2013; Brankovics et al., 2018). Despite the relatively conserved gene content, genome structural changes caused by insertion of repetitive elements, gain or loss of introns, and/or gene transfers to the nucleus often lead to mitogenome size variations (Burger et al., 2003).

Introns are present in many fungal mitogenomes. These sequences can be divided into two groups: group I and group II. Introns Group I (IGI) presents six subclasses and are more abundant in mitogenomes than the group II. IGI can encode cellular proteins, such as *rps3* ribosomal protein or maturases like homing endonucleases (HEGs), which are likely involved in the splicing process (Hausner, 2003). IGI are located within genes and are associated with shaping of mitogenomes, gene rearrangements and also in the host fitness (Hausner, 2003; Belfort, 2017). Introns type II are self-splicing and consist in a sequence of approximately 600 nucleotides. Usually, they are similar to IGI and can be classified into four groups (Michel et al., 1989; Hausner, 2003).

A recent study (Sandor et al., 2018) based on 20 species/groups of fungi revealed, for most of them, higher genetic diversity of nuclear genes and genomes than for the mitochondrial ones. This pattern is more similar to the observed in plants but different from most of the animals. The mechanisms responsible for the structural variations in fungal mitochondrial genomes and its relationship with fungi divergence time are still poorly understood. Nevertheless, the increasing availability of fungal mitogenome sequences in public databases can help us to understand the factors that drive fungi mitogenome variations of fungi.

The order Hypocreales (Ascomycota) is a monophyletic group with more than 2700 species of fungi, distributed in 240 genera divided in sexual (teleomorph) and asexual (anamorph) morphs (Varshney et al., 2016; Wijayawardene et al., 2018). Organisms of this order show a great variability of ecological functions and may display roles ranging from saprobes to pathogens. Special interest has been given to economically important species in the fields of pharmacology and medicine, biological control and biotechnology (Rehner and Samuels, 1995; Varshney et al., 2016), such as *Trichoderma harzianum*. This species is widely distributed in soil and plants due to its diversity of ecological niches (Druzhinina et al., 2011; Contreras-Cornejo et al., 2016). Furthermore, *T. harzianum* acts as a biofertilizer, promoting plant growth, and as a biocontrol agent during plant infections (Druzhinina et al., 2011; Harman, 2011), and has been considered an attractive alternative to the usage of chemical fungicides (Schmoll et al., 2016). Despite its economic relevance and wide distribution, the nuclear genome of *T. harzianum* is not yet fully assembled, and the mitochondrial genome is still not available in public databases.

Herein, we performed the deep sequencing of *T. harzianum* HB324 mitochondrial genome and compared it with 34 other reference species from the Hypocreales order that were available in public databases. We performed *de novo* structural and functional annotation of all species analyzed. The standardized annotation allowed us to perform comparative analyses revealing high variation among the mitogenomes, even within individuals from the same family. Mitochondrial genome length varied from 24,565 to 103,844 bp. Despite the size variation, the copy number, size, and structure of protein-coding genes were highly conserved, suggesting that differences in the genome length are likely related to non-coding regions, such as introns, HEGs and unidentified ORF (uORF). Introns were the most widespread non-coding element found in mitogenomes. Analyses based on sequence similarity have revealed greater sharing of intronic sequences within families. In some cases, we observed conservation even in the position of intron insertion, such as the intron within gene *rrnL*, present in almost all species. In contrast, species with higher divergence time showed smaller similarity among intronic sequences. We also investigated whether the genetic divergence between fungal species would be positively correlated with the size of the mitogenome and the prevalence of protein-coding and non-coding mitochondrial elements. Fungi species that evolve faster presented a higher frequency of non-coding elements on their mitogenomes. Therefore, our data suggests that the difference in the size of the mitogenomes is due to the presence of non-coding components. Besides, species that evolve faster have a greater proportion of non-coding elements, corroborating their role in the shaping of fungi mitochondrial genomes.

## Materials and Methods

### Sequencing, Assembly and Annotation of the Mitochondrial Genome of *Trichoderma harzianum* HB324

Pure culture of *T. harzianum* HB324 isolate was grown on 2% malt extract agar (MEA) for 5 days. The mycelium was collected from the agar surface and transferred to a 2 mL tube containing lysis buffer. DNA extraction was performed according to the instructions of FastDNA kit (MP Biomedicals, CA, United States). The quality and quantity of genomic DNA were assessed by electrophoresis and fluorometric analyses using Qubit dsDNA BR Assay Kit (Thermo Fisher Scientific, Waltham, MA, United States). The sequencing library was prepared using the NEBNext Fast DNA Fragmentation and Library Preparation Kit (New England Biolabs, Ipswich, NE, United States) according to the manufacturer’s instructions. The library was sequenced on a HiSeq 2500 sequencer (Illumina, San Diego, CA, United States).

Quality of reads was assessed using FastQC v0.11.5 program^1^. Adapter sequences and bases with Phred score < 20 were removed using BBduk software from BBtools package^2^. After trimming, sequences were assembled using the software SPAdes v 3.11.1 software (Bankevich et al., 2012). The identification of mitochondrial sequences was performed using similarity searches against reference mitochondrial genomes deposited in NCBI RefSeq database using BLASTn (Altschul et al., 1990). The contigs with the highest coverage scores were selected for subsequent annotation. The raw data were deposited on NCBI SRA database under the accession number PRJNA604815.

Annotation of mtDNA was performed using MITOS2 software^3^, with the NCBI fungi RefSeq 81 database as reference and using the genetic code 4 (Mold, Protozoan, Coelenterate Mitochondrial Code) for CDS translation. The softwares MFannot and RNAweasel^4^ were used for annotation of intronic regions and uORF sequences. The introns identified in the *T. harzianum* mitogenome have been named according to the nomenclature proposed by Johansen and Haugen (2001) and Zhang and Zhang (2019). Repetitive DNA sequences were identified using uGene (Okonechnikov et al., 2012), and a circular mitogenome map of *T. harzianum* HB324 was generated using the output annotation file of Geneious v9.1.6^5^. Since mitogenomes from Hypocreales order are circular, we verified the completeness of *T. harzianum* mitogenome by amplifying the *rrnl* ribosomal gene that presented three exons, one at the beginning (position 1591 – 2191) and other at the end (position 30158-30370) of the assembled genome. The oligonucleotides used to perform the amplification were: rrnl_F 5′ TTGTTGCACTAATCTCCGAA 3′ and rrnl_R 5′ ATTGCATCTTGTGATCCTGT 3′. PCR products were purified and sequenced^6^ (Belo Horizonte, Brazil) on an ABI 3130 automated sequencer (Applied Biosystems, Life Technologies Q7, CA, United States).

### Comparative Analysis of the Mitogenomes of Hypocreales Order

Comparative analyses were performed using the assembled mitogenome of *T. harzianum* HB324 plus 34 reference mitogenomes of species of the order Hypocreales publicly available on NCBI Organelle Genome Resources database^7^. Identification of fungal species and accession numbers for their mitochondrial and nuclear genomes are provided on Supplementary Table S1.

The 34 mitogenomes retrieved from public databases were reannotated using the software MITOS2^3^, RNAWeasel and MFannot^4^ to standardize the annotation and thus, allowing the comparative analysis. The resultant annotation file of each mitogenomes was submitted to manual curation taking into account number and presence of core genes and tRNAs. Gene duplication was confirmed by comparative analysis using sequence similarity among putative duplicated sequences. Duplication was considered when target sequences presented both identity and coverage of at least 50%. The mitogenome characterization included analyses of fungal mitogenome length, gene copy numbers and integrity, presence of introns, homing endonucleases, coding and non-coding regions, *tandem* repeats, unidentified ORFs (uORFs), as well as GC content. We developed in-house scripts to parse software outputs and calculate genome stats (Supplementary Text S1). In case of duplicated genes, it was estimated whether the two copies have coding potential using CPC2 software (Kang et al., 2017). Coding regions were considered as those regions composed by protein-coding genes, ribosomal and tRNA sequences. The non-coding regions were defined as the regions composed by intergenic, intronic, uORFs and HEGs sequences. Correlation analyses were performed using *Pearson* correlation in order to verify whether mitogenome length and number of annotated genomic elements were responsible for a positive association, such as number of protein-coding genes, introns and HEGs. All the analyses were performed using R software (R Core Team, 2013) and the packages hmisc (Harrell, 2019), gplots (Warnes et al., 2019), ggplot (Wickham, 2016), and reshape (Wickham, 2018). The mitochondrial gene ordering was determined using an in-house script and considering the ribosomal gene *rrnL* as the first element.

### Sequence Similarity and Conservation of Intronic Sequences

Sequence similarity analyses were performed for all the introns identified in the 35 species investigated in this study according to the methodology proposed by Pogoda et al. (2019) with minor modifications. Similarity searches were performed using BLASTn from Blast package (Altschul et al., 1990) and sequences with coverage and identity higher than 60 and 50%, respectively, were considered similar. It was also established if there was sequence similarity between introns of the same species, which could be indicative of intron duplication. Intron similarity was based on sequence identity and was visualized using R software with circlize package (Gu et al., 2014) and correlation plots, which were performed in R software with ggplot and corrplot packages (Wickham, 2016; Wei and Simko, 2017). Intron conservation was evaluated based on the intronic sequence insertion positions on the target gene. In this case, the introns located within genes were aligned into its respective gene and it was verified if the position of the insertion was conserved among the same gene in different species, as demonstrated by Deng et al. (2016) and Zubaer et al. (2018). In our analysis, we considered the position of insertion as a region composed of 11 nucleotides that precede the intron, allowing only one nucleotide of mismatch. The data were plotted using the R software.

### Transfer of Mitochondrial Genes to the Nuclear Genome

The presence of fungal mitochondrial genes in the nuclear genome was assessed by sequence similarity searches using Blast software according to Li et al. (2019) with some modifications. For this analysis, 18 nuclear fungal genomes publicly available to date (out of the 35 evaluated in this study) were used (Supplementary Table S1). For each of the species evaluated, the protein-coding genes were translated into amino acid sequences and ribosomal gene (nucleotide sequences) were compared with the nuclear genome. Transference of mitochondrial genes to the nuclear genome (NUMT) was considered when the two sequences presented at least 50% of similarity and coverage with *E*-value < 1e-10.

### Evolutionary Analyses

Genes exclusively found in the mitogenomes were used for molecular clock analyses. For each species, we concatenated the gene sequences of *atp8*, *atp9*, and *cox3*. Since these genes were not present in any nuclear genome evaluated, they were used to calculate the synonymous substitution rate (dS) for each species. Sequences were aligned by MAFFT (Katoh et al., 2017) and submitted to the web platform SNAP^8^. The results of dS were compiled and for each species was estimated the $\bar{d\,S}$ based on the formula: $\sum \frac{d\,S}{n-1}$ was estimated. We calculated *Pearson* correlations between the $\bar{d\,S}$ from the last common ancestor for each pair of species with the size of protein-coding genes, introns, HEGs, uORFs, and the genome size. These analyses were carried out with the ggplot package on R. From the alignment of *atp8*, *atp9*, and *cox3* genes, a time tree scaled analysis was also performed. The selection of the best nucleotide substitution model was made using MEGA X based on AIC criteria (Akaike, 1974). The Maximum Likelihood tree with 1000 replicates using the model GTR G+I was built in the same software. RealTime-ML analyses were carried out using the GTR G+I model to create the evolutionary time-based tree. Three ancestral nodes were used for time calibration: the common ancestor node between the order Hypocreales and *Neurospora crassa* (outgroup) ranging from 314 to 414 Mya (Van der Nest et al., 2015), the common ancestor of the family Nectriaceae with *Acremonium chrysogenum* (*incertae sedis* - Hypocreales) ranging from 246 to 294 Mya (Sung et al., 2008), and the common ancestor of the families Hypocreaceae, Ophiocordycipitaceae, and Clavicipitaceae ranging from 162 to 168 Mya (Yang et al., 2012).

## Results

### Deep Sequencing of *T. harzianum* mtDNA Reveals Variance Within *Trichoderma* Genus

**FIGURE 1:**
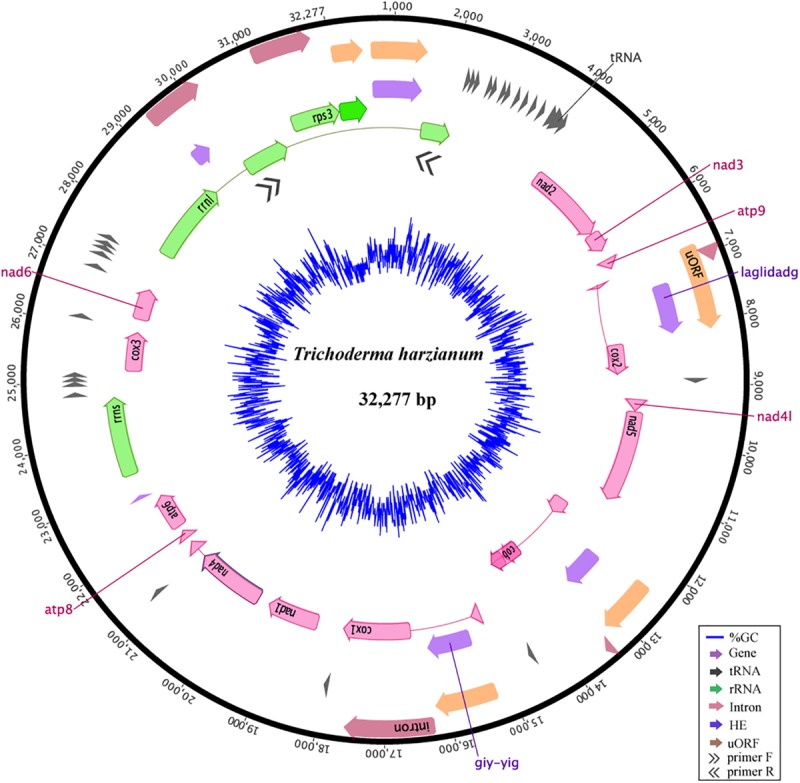
Mitochondrial genome of the *Trichoderma harzianum* isolate HB324. Homing endonucleases and introns are represented in purple and red, respectively. Ribosomal genes are shown in green, tRNA encoding genes are represented in gray, ORFs without defined function (uORF) are colored in orange and core genes are highlighted in pink. Elements connected by thin lines represent fragments from the same gene. The blue ring represents the GC content. Double arrows represents the primers and region amplified in the gene *rrnl*.

The mitogenome of *T. harzianum* isolate HB324 is a circular DNA molecule of 32,277 bp (Supplementary Text S2), GC content of 27.74%, composed of 14 genes related to the oxidative phosphorylation (*atp6, atp8, atp9, cox1, cox2, cox3, cob, nad1* – *nad4, nad4L, nad5, nad6*), 28 genes encoding for transfer RNAs, two ribosomal RNAs [one encoding to the small subunit (*rrns*) and other for the large subunit (*rrnl*)] and the ribosomal gene encoding to *rps3* protein. The protein-encoding region represented 52,23% of the genome, while non-coding elements cover up 22,37% including four uORFs (or hypothetical genes), five introns IGI and six homing endonucleases (HEGs). All the features were encoded in the same DNA strand (Figure 1). The *rnl* gene amplification confirmed that the mitogenome was complete and is a circular molecule (see primer positions represented by double black arrows in Figure 1). Detailed information of features annotation is available in Supplementary Table S2.

**FIGURE 2:**
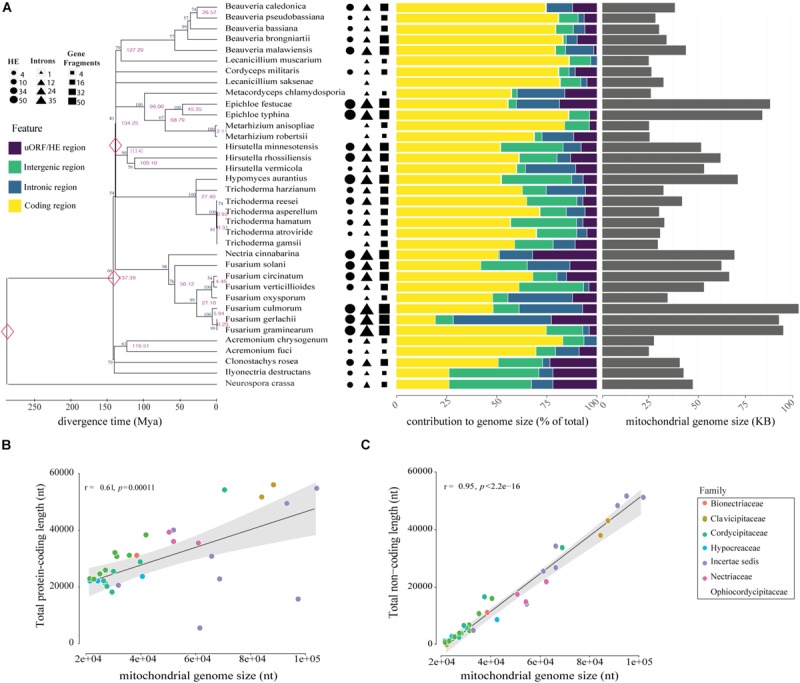
Genomic and phylogenetic characterization of the 35 mitogenomes analyzed in this study. **(A)** Timetree analysis, mitogenome composition (%) and genome size (nt) of species evaluated are shown on the left, middle and right panels, respectively. In the analysis of genomic content, yellow represents protein-coding regions, purple represents uORF and HEGs, introns are showed in blue and intergenic region in green. Genomic data was normalized according to the mitogenome size of each species. Correlations between mitogenome size and protein-coding **(B)** or non-coding content **(C)**. Correlation was computed using Pearson test.

Compared to other *Trichoderma* mitochondrial genomes, *T. harzianum* has the second largest genome and the highest number of IGI sequences within genes (5) (Figure 2A). The species *T. reesei* has the largest mtDNA, even with a smaller number of fragmented genes when compared to *T. harzianum* (4); however, the IGI size accounts for almost 25% of its genome size. In contrast, *T. gamsii* and *T. asperellum* have the smallest mitochondrial genomes (Figure 2A). The number and size of IGIs in both species represented only 5 and 10% of their mtDNA, respectively. Since it was observed a considerable variation in the structure and size of mitogenomes even within the same genera, such as *Trichoderma*, we decided to extrapolate our investigation to all the families from Hypocreales order (Figure 2A).

### Comparative Analyses Reinforce Discrepancies Among Mitogenomes of Hypocreales Order

The comparative analyses of the 34 mitogenomes from species of Hypocreales order and the mitochondrial genome of *T. harzianum* sequenced in this study revealed high variation in mitogenome length and content. The mitogenome sizes ranged from 24,565 (*Acremonium fuci)* to 103,844 bp (*Fusarium culmorum*), with a mean value of 47,492 bp (Figure 2A). The largest genomes exhibited the largest intronic regions (for instance, *Fusarium gerlachii, F, culmorum* and *F. graminearum*) and the smallest encoding regions (remarkable to *F. solani* and *F. graminearum*). Small genomes are less occupied by introns, or they are absent, such as in *Metacordyceps chlamydosporia*, *Metarhizium anisopliae*, and *M. robertsii.* The absence of uORFs was also observed in genomes of reduced size, for example, *Beauveria pseudobassiana, B. bassiana, Lecanicillium muscarium, F. oxysporum* and *Acremonium fuci* (Figure 2A).

The correlation analyses indicated that genome size is somehow correlated with the total length of protein-coding region (Figure 2B). Nonetheless, length of non-coding regions showed a higher and more significant correlation with the size of mitogenomes (Figure 2C). Other non-coding elements, such as uORFs and tandem repeats, also showed a positive and significant correlation with the mitogenome size (Supplementary Figure S1). Nevertheless, mitogenome length did not correlate with evolutionary history of the species, suggesting that the structure and content are prone to recent and fast evolutionary changes (Figure 2A). Supplementary Table S3 provides the size, number of mitochondrial genes, uORFs, introns, GC content, and repetitive elements for each of the mitogenomes analyzed.

**FIGURE 3:**
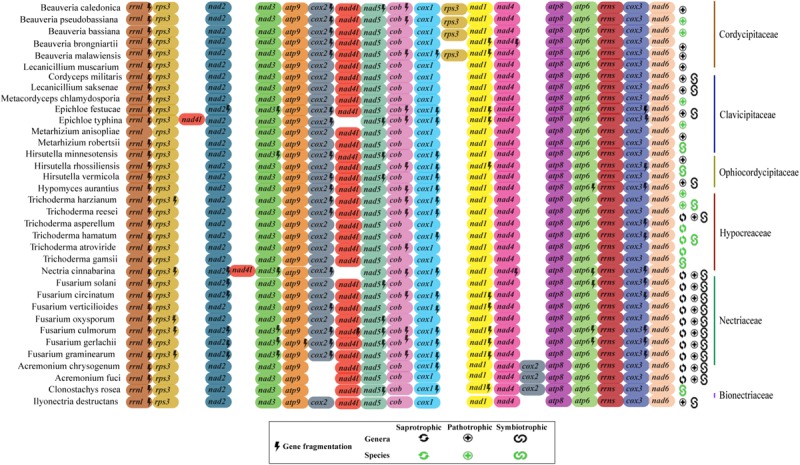
Genomic ordering of the 17 core elements present on mitochondrial genomes. Were analyzed 14 protein-coding genes, two ribosomal genes and the *rps3* protein from the 35 fungal species investigated in this study. Each gene is indicated by a different color. Genes that showed more than one fragment are marked with a lightning (fragmented genes). Classification of the species at family level is shown in the right. The ecological function (saprobic, pathogen, and symbiotic) of each species is also shown. In green it is represented the ecological function at species level and in black at genera level.

Although we observed correlation between the coding region and mtDNA length, the structural analysis of the 17 mitochondrial genes (14 protein-coding genes, two rRNA and one *rps3*) of the 35 fungal mitogenomes revealed that Hypocreales mitogenomes display high conservation. Nonetheless, variations were found in the genomes of the species *Acremonium chrysogenum, A. fuci*, and *Clonostachys rosea*, in which the position of the *cox2* gene is displaced. The species *Epichloe typhina* and *Nectria cinnabarina* presented a change in the position of the gene *nad4L*. Additionally, the four species of the genus *Beauveria* showed two copies of the gene *rps3* (Figure 3). This was the only gene for which two copies of a quite similar size were detected. One copy was located within the *rrnl* gene and the other is freestanding, situated between *cox1* and *nad1* genes. The freestanding gene copies are presumably able to encode to proteins (active), whereas those located within the *rrnl* seems to be disrupted, with exception for the genes of *Beauveria caledonica*, as no coding potential was detected for both copies. Still, although we noticed differences in the ordering and copy numbers of protein-coding genes among Hypocreales mitogenomes, our analyses revealed low variation regarding the structure and genic content representativeness, pointing out that the differences observed among mitogenomes mainly accumulate on non-coding regions (Figure 3 and Supplementary Figure S2).

### Diversity of Non-coding Elements

Non-coding elements were found in all mitogenomes evaluated. The representativeness of these elements oscillated from 3% in *Lecanicillium muscarium* to 48% *in Fusarium graminearum*. The content of these elements varied among and within families, suggesting that it is not only related to phylogenetic relationships (Figure 2A). Among the non-coding elements, we identified IGIs containing uORFs and HEGs. Six types of introns were found in the analyzed mitogenomes, five types classified as elements of group I and one type belonging to group II. In the total, 349 introns were identified, out of which 210 had a putative uORF. Besides, 450 HEGs were identified and classified according to their genomic origin as freestanding or located within intronic sequences. The most frequent type of HEG was LAGLIDADG (63.78%), followed by GIY-YIG, that was identified in 36.2% of the HEG occurrences. The species with the highest number of HEGs was *Fusarium graminearum*, with 32 occurrences. *Epichloe festucae* exhibited the highest number of uORFs (46), while the highest number of introns was identified in *Fusarium culmorum*, totalizing 39 elements (Figure 2A).

### Distribution and Evolutionary Conservation of Intronic Sequences in the Mitochondrial Genes

**FIGURE 4:**
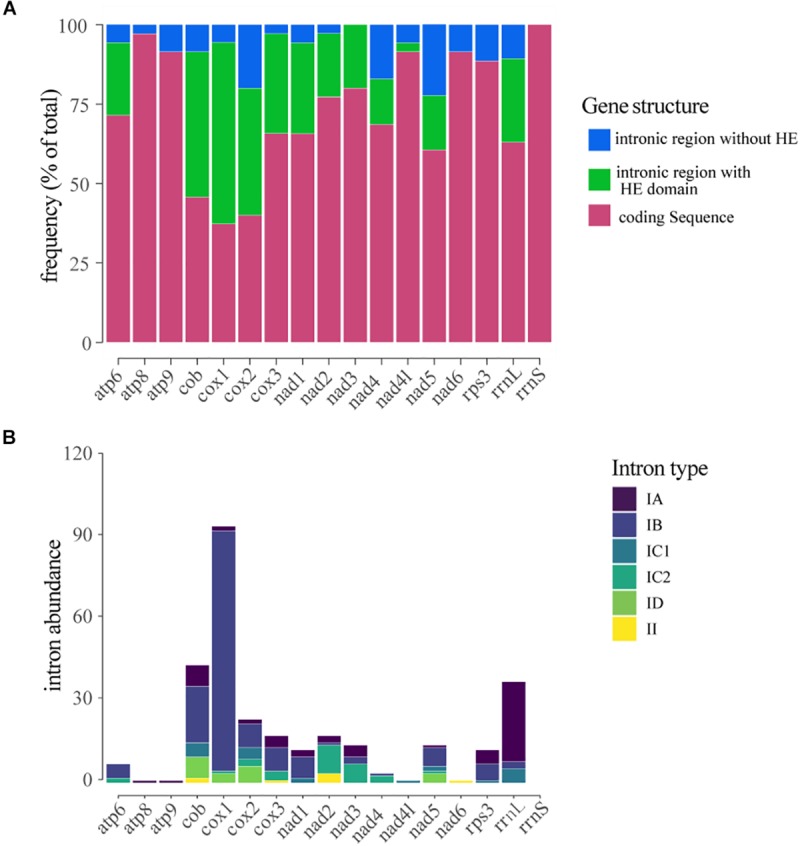
Distribution and classification of introns located within mitochondrial genes. **(A)** Frequency of introns and/or homing endonucleases located within the gene. **(B)** Classification of the introns detected in each gene.

The number of IGIs detected in the mitochondrial genes varied significantly. Considering all the introns identified in the mitogenomes, the genes that showed the highest number of IGIs were *cox1* (109 IGIs), followed by *rrnl* (43), *cob* (50), *cox2* (27), *cox3* (20) and *nad2* (20). In contrast, we did not find any occurrence of IGIs within *rrns* ribosomal gene. The introns located within genes *cox1* (57%), *cob* (45%), and *cox3* (31%) exhibited the highest frequency of sequences containing HEGs (Figure 4A). The number and size of IGIs correlated positively to the number and size of HEGs, suggesting the HE could have a role in IGIs spread, and consequently, in the length and structure of the mitogenomes (Supplementary Figure S3).

Most of the introns found in this study were classified as group I, mainly type IB (208 – 59,6%) while only eight were classified as group II introns. The type IA intron showed the largest abundance and stood out for its average size of 1,000 bp, while the other introns presented a mean size of ∼250 bp. Group I introns were detected in all genes analyzed; however, in the genes *atp6*, *cob*, *cox1*, *cox2*, *cox3*, and *nad1* the type IB intron was the most common; in the genes *rrnl*, *rps3* and *atp9* type IA introns were prevalent; in the genes *nad2*, *nad3*, and *nad4* the type IC1 intron showed higher frequency, and in *nad4l* gene only the type IC2 intron was detected. Group II introns were only detected in the genes *cox1*, *cox2*, *cox3*, and *nad2* (Figure 4B).

**FIGURE 5:**
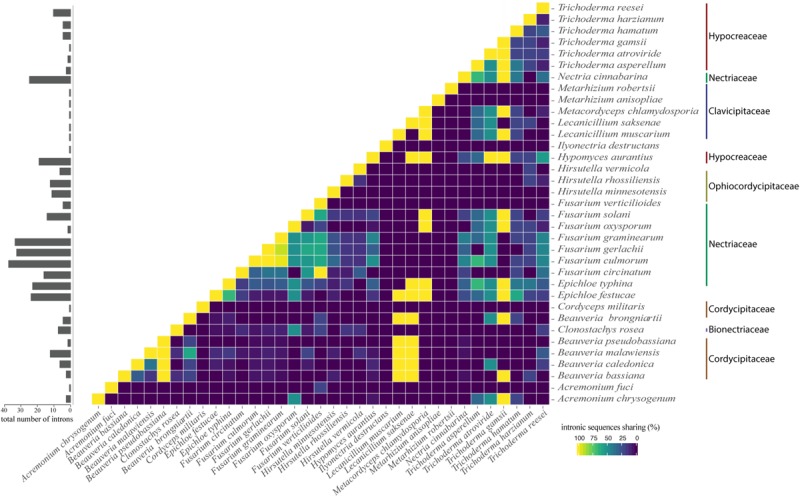
Correlation plot showing profile of intron similarity among species. The number of introns for each species is shown in the bar chart on the left panel. The value was normalized by comparison with the species that showed the largest number of introns.

**FIGURE 6:**
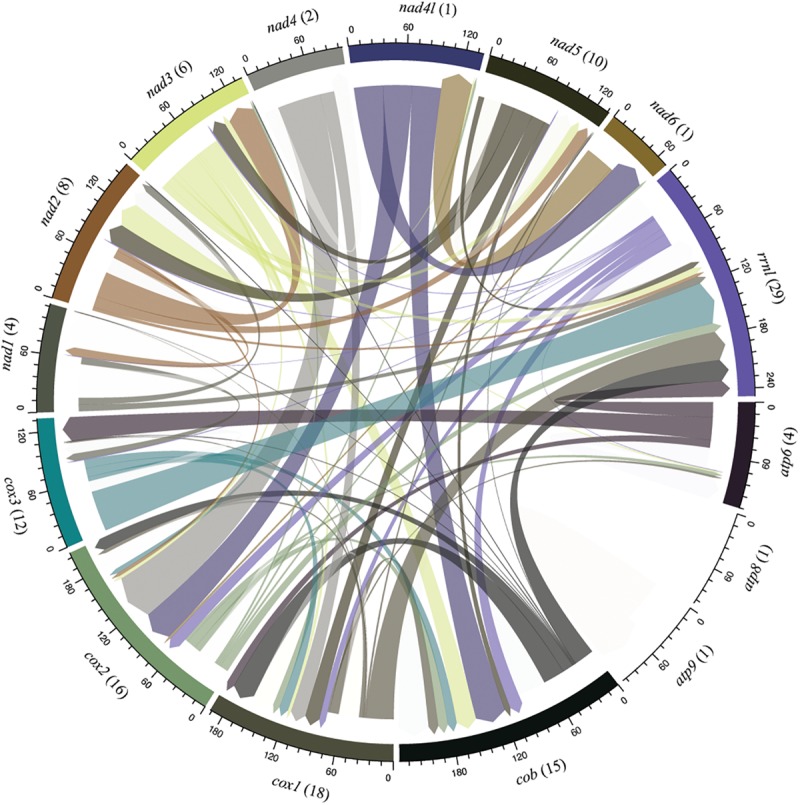
Sharing profile of group I introns according to the mitochondrial gene origin. Circular plot indicates intron sharing pattern based on sequence similarity among core elements of the mitochondrial genomes. Each gene is represented by a colored segment. Introns that are shared with more than one gene (translocation) are shown in different color. Introns shared among species located within the same gene are not shown in the figure and were investigated in the **Figure 7**. The number of introns shared in each gene are indicated in parenthesis. Introns from the genes *atp8* and *atp9* present sequence similarity only with introns identified in its respective gene.

Interestingly, we noticed sequence similarity among intronic sequences from different species. High similarity among IGI sequences was observed among species of the same family as, for example, *F. graminearum*, *F. culmorum*, and *F. gerlachii* which shared 27 introns sequences (Figure 5). We also noticed similarity among IGIs sequences from different families, such as for Hypocreaceae, Cordycipitaceae, Clavicipitaceae, and Nectriaceae (Figure 5). The IGI sequence identified in the *rrnl* gene was the most widespread element within mitogenomes from Hypocreales order, displaying high similarity with introns located within the same gene and other different genes, such as *atp6*, *cob*, *cox1*, *cox2*, *cox3*, *nad1*, *nad2*, *nad3*, *nad5*, and *rps3*. This profile suggests that the conservation of IGI sequences among genes could have originated from a common ancestor (Supplementary Figure S4). Other examples of IGI sequence sharing are notable, such as for *cox1* and *cob*; *cob* and *cox2*; *nad2* and *nad3*; *nad3* and *nad5*; *nad4l* and *nad6; atp6* and *cox3* (Figure 6). Despite conservation of many IGIs, such as those located within the genes *rrnl*, *cox1*, and *cob*, we identified IGIs that are not shared and figure as specific to some species, suggesting that these sequences could be acquired by different mechanisms, such as horizontal transference (Supplementary Figure S4).

**FIGURE 7:**
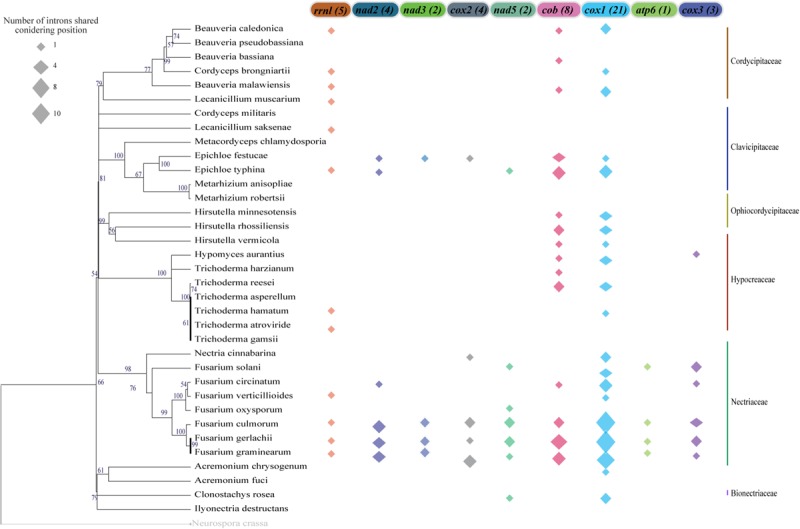
Group I Intron sharing pattern considering insertion sites on the mitochondrial genes. For the insertion point that is common in more than one species, the position was considered conserved and it was shown in the figure. The total number of sharing events are indicated in parentheses next to each gene name. The size of the polygon represents the number of sharing events in each gene and species. The mitochondrial genes that are not represented in the figure do not present any sharing position event or do not present intron sequences.

We also investigated the conservation of intronic sequences taking into account the insertion site and gene which it is located. In this context, we were also able to detect IGI sequences that were conserved among species, although the conservation was always restricted to the maximum of 50% of the analyzed species (Figure 7). Nevertheless, closely related species showed higher rates of IGI sharing based on position. For instance, some species of the genus *Fusarium* shared, with at least one other species, IGIs in almost all the mitochondrial genes evaluated. The gene *cox1* presented the highest number of shared IGIs per position, with 21 events. Besides, the *nad3* and *nad5* genes were the genes with the lowest numbers of conservation events (2 introns in each gene) (Figure 7 and Supplementary Table S4).

### Transfer of Mitochondrial Genes to Nuclear Genomes

Our results showed that mitochondrial genomes have numerous HEG elements, which are capable of self-mobilization (Hausner, 2003). Since HEGs can interrupt or structurally modify mitochondrial genes impacting on mitogenome evolution, we evaluated the duplication of mitochondrial genes to the nuclear genome. We investigated the presence of the 14 mitochondrial protein-encoding genes and the ribosomal genes in nuclear genomes of 18 fungal species belonging to Hypocreales order available in public databases. Except for *atp8*, *atp9*, and *cox3*, all the other mitochondrial genes were found duplicated, in at least one species, in the nuclear genomes. The genes *nad5* and *cob* showed the highest frequencies of duplication in the nuclear genomes (three events each), followed by *cox1*, *rrnl, nad1, and nad2* (two copies each) and *rrns*, *rps3*, *nad3*, *nad4L*, *nad4*, *atp6*, and *cox2* with only one event of transference to the nuclear genome.

Regarding the number of events in the same species, *Fusarium graminearum* had four genes (*nad6*, *atp6*, *cob*, and *nad5*) with copies in both nuclear and mitochondrial genomes, while the species *Trichoderma harzianum* (*nad2*, *cob*, and *cox1*) and *T. gamsii* (*nad1*, *nad3*, and *nad5*) had three genes that were possibly transferred to the nuclear genomes. The species *Metacordyceps chlamydosporia*, *Cordyceps militaris*, *Fusarium circinatum*, *F. verticillioides*, *F. oxysporum*, *Trichoderma asperellum*, *Epichloe typhina* and *Clonostachys rosea* did not show any event of duplication of mitochondrial genes to the nuclear genome (Supplementary Figure S5).

### Relationship Among Divergence Time and Coding and Non-coding Elements on Mitogenomes

**FIGURE 8:**
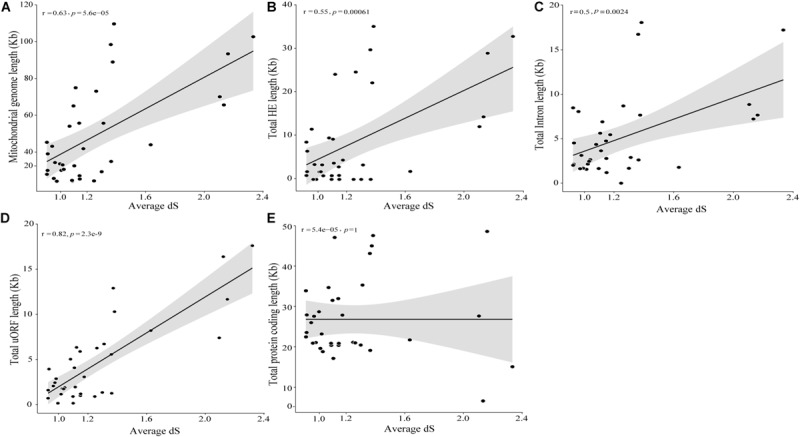
Correlation among divergence time and mitochondrial genome characteristics. Correlation analyses among divergence time and mitochondrial genome **(A)**, intron **(B),** HEGs **(C),** uORF **(D)** and protein coding **(E)** length.

The timed phylogenetic analyses based on the sequences of the genes *atp8*, *atp9*, and *cox3* showed that the order Hypocreales evolved over a period of 137.39 Mya. The species *Ilyonectria destructans* and *Clonostachys rosea* diverged ∼120 Mya, while the species *Fusarium graminearum* and *F. gerlachii* diverged recently, ∼0.23 Mya. Assessment of the estimated time-tree suggested that the presence of introns may not be related to phylogenetic relationships (Figure 2A). Nonetheless, there was a significant correlation among the average rate of substitutions at silent sites of each species $\bar{d\,S}$ and the mtDNA size, indicating that evolutionary time have a role on mitogenome shaping (Figure 8A). Moreover, there was a significant correlation between $\bar{d\,S}$ and total length of introns (Figure 8B), total length of HEGs (Figure 8C) and total length of uORFs (Figure 8D). On the other hand, we did not observe correlation among $\bar{d\,S}$ and the total length of protein-coding regions (Figure 8E), indicating that fast-evolving mitogenomes tend to accumulate evolutionary changes in non-coding regions.

## Discussion

Comparative genomics has been used to elucidate the population structure and to understand the functions of each species in an environment (Nadimi et al., 2016). In the last years, this methodology has revealed the main genomic organization of fungal species, factors related to fungal pathogenicity, production of substances and enzymes of commercial interest, and other aspects of fungal evolution and biology (Stajich, 2017). Mitogenomes are studied for phylogenetic relationships, phylogeography and structure dynamics in the population since their genomes present conserved gene content, rapid evolutionary rate and many copies in the host‘s cell (Moore, 1997). Herein, we present a new mitochondrial genome of *Trichoderma harzianum* and performed comparative analysis including 34 species from Hypocreales order to investigate variations in the mitochondrial genome size and structure, intron sharing, presence of NUMTs, and the relationship among divergence time and coding and non-coding elements that compose these mitogenomes.

*Trichoderma* species are found in almost every environment, mainly in soil and plant root ecosystems (Harman et al., 2004) and are widely applied in agriculture as biocontrol agents, inhibiting the growth of other fungi and nematodes (Lorito et al., 2010). Furthermore, some species can stimulate plant growth and development by stablishing mutualistic beneficial interactions (Bonfante and Requena, 2011). The species *T. harzianum* is widely used in agricultural industries due to its mycoparasitic activity, which inhibits many plant pathogens (Lorito et al., 2010). *T. harzianum* HB324 was isolated by our research group from *Hevea brasiliensis* (rubber tree) leaves and our data have shown that this isolate can act as biocontrol agent against the phytopathogen *Colletotrichum* sp. (Fonseca et al., 2018). Despite the economic and ecological importance of this fungus, its mitochondrial genome has not been yet available. In the current work, the complete sequencing of mitochondrial genome of *T. harzianum* HB324 isolate is presented, and its mitogenome has 32,277 bp, being the second largest of the genus and contain four uORFs, five introns and six domains of HEGs.

The remarkable variation in the mitogenomes sizes of the 35 fungal species evaluated in this study (from 24 to 103 Kb) was observed at both genus and species levels: within strains of *Trichoderma*, *Beauveria*, and *Fusarium*, for example, it reached up to 70 Kb. These results reinforce those previously reported. There are studies describing genomes of around 18 Kb in *Hanseniaspora uvarum* (Pramateftaki et al., 2006) and up to 235 Kb in *Rhizoctonia solani* (Losada et al., 2014). Variations in mitogenome sizes at genus and species level have also been reported within species of the genera *Schizosaccharomyces* (Bullerwell et al., 2003) and *Fusarium* (Al-Reedy et al., 2012), as well as within the species of *Saccharomyces cerevisiae* (Wolters et al., 2015), *Cordyceps militaris* (Zhang et al., 2015), and *Rhizophagus irregularis* (Formey et al., 2012).

The wide size range of the mitogenomes evaluated in our study may be explained in part by variations in length of intergenic regions, and differences in number and length of introns. The presence or absence of introns have been widely described as the major contributing factor for such variation among fungi (Jung et al., 2010; Joardar et al., 2012; Zhang et al., 2015). Among the fungal strains evaluated in our study, *F. graminearum* had the highest number of introns (35), accounting for 54% of the entire mitochondrial genome. Our results are similar to those reported to *Podospora anserina* (Ascomycota), for which 33 mitochondrial introns were detected (Cummings et al., 1990), as well as to the Basidiomycota mushroom *Agaricus bisporus*, for which 45.3% of the mitochondrial genome was composed of introns (Ferandon et al., 2013). Our analysis also revealed fungal species with no intron sequences, such as *Metarhizium anisopliae* and as previously described for *Mycosphaerella graminicola* (Torriani et al., 2008).

In fungal mitogenomes two classes of introns are found, group I and/or group II. They can be distinguished from each other by their sequence, structure and splicing mechanisms. Group I has been reported as the most widespread in fungal mitogenomes and can be subdivided into IA, IB, IC1, IC2, IC3, and ID (Saldanha et al., 1993; Hausner, 2003; Lang et al., 2007). Mitochondrial introns can self-disperse in mtDNA, acting as mobile elements due to the presence of enzymes that are able to cleave double-stranded DNA to insert introns into new genomic locations (Lazowska et al., 1980; Pellenz et al., 2002). HEGs are encoded by group I and II introns or can be found freestanding. They are classified into six families, but only two are detected in fungal mitogenomes, LAGLIDADG and GIY-YIG (Stoddard, 2014).

In our study, we identified all group I subgroups of introns, except IC3, and a few sequences of the group II. The most common intron type was IB (59.6%) and IA (17.2%), while group II introns were the least frequent, and accounted for only 2.3% of the intron sequences identified. Furthermore, 450 HE sequences, 275 LAGLIDADG and 175 GIY-YIG, were detected. Our data are corroborated by many studies, such as that published by Zubaer et al. (2018), who described the presence of 81 introns, 72 of which containing HE, in the fungus *Endoconidiophora resinifera*. Most of the introns detected (32) were classified as group IB, while only three introns group II were encountered. Moreover, the HEG of the LAGLIDADG family was the most frequent. In *Ophiocordyceps sinensis*, 52 introns were found, of which only six were classified as group II, and subgroup I was recovered as the most frequent (Li et al., 2015).

Group I introns (IGI) are considered the largest class of introns. Introns from this group are mostly self-splicing or are spliced by protein factors that stabilize the intronic RNA that undergoes conformational changes, such circularization. This type of intron can also have repetitive sequences or protein coding sequences at the ends of the intron, as seen in the case of the *rps3* ribosomal protein gene (Nielsen and Johansen, 2009). In addition, some introns have been described to be involved in rRNA and tRNA folding (Cao and Woodson, 2000; Rangan et al., 2004), suggesting that introns are important regulators of the expression of genes encoding proteins and ribosomes in mitogenomes (Nielsen and Johansen, 2009). IGIs may also be important for the fitness of the fungal host. Intron group I colonized by HEGs can promote RNA splicing, due to the presence of maturase which acts as a cofactor promoting the splicing between the intron and the precursor RNA (Lambowitz and Perlman, 1990; Belfort, 2003). IGIs may have their splicing rate altered due to exposure to certain environmental factors (Lambowitz et al., 1998). For example, in chloroplasts, it has been reported that type IGI splicing was activated by light (Deshpande et al., 1997). Splicing inhibitory factors have also been described, such as the molecular Flavin mononucleotide (FMN), which directly interferes with the affinity of the intron with molecules involved in the catalysis of the intron itself (Kim and Park, 2000). Since growth conditions such as temperature, luminosity, pH and salinity have already been described influencing the rate of splicing in other organisms, it was suggested by Belfort (2017) suggested that some IGIs can work as biosensors when exposed to certain environmental factors, promoting a selective advantage over the intronless fungi. Fungal species such as budding yeast and *Neurospora crassa*, which do not present introns have been described presenting respiratory defects (Lambowitz and Perlman, 1990; Hausner, 2003). Additionally, type I introns have been studied to be used as ribozymes that have the ability to inactivate specific regions of the host itself or viral sequences based on the messenger RNA (mRNA) cleavage (Johansen et al., 1997; Lambowitz et al., 1998).

Our data also revealed a positive correlation between the number and length of IGIs and HE. This has also been reported for the fungus *Agaricus bisporus* (Ferandon et al., 2013) and *E. resinifera*, for which most of the introns contained HE domain, and 50% of intron size was due to the presence of HEGs (Zubaer et al., 2018).

Introns were distributed all over the mitochondrial genes evaluated, and *cox1* was the main introns reservoir, followed by *rrnl*, *cox2*, *cob*, *cox3*, and *nad2*. According to Ferandon et al. (2013) and Zubaer et al. (2018), *cox1* is the main intron reservoir in fungal mitogenomes. The authors reported the presence of 19 introns in *A. bisporus* and 23 introns in *E. resinifera*, which was considered the fungal species with the largest number of introns in this gene. Introns have also been identified in other genes, such as *cox2*, *cob*, and *rrnl* in two species of *Rhizopogon* (Agaricomycotina) (Li et al., 2019). In the fungus *O. sinensis* (Hypocreales), the genes *cox1* and *rrnl* were reported as the main reservoir of introns (Li et al., 2015). Moreover, in the fungus *Stemphillium lycopersici* the putative *cox1* and *cox2* gene fusion accounted for nearly half of the number of introns encountered in the mitochondrial genome (Franco et al., 2017).

It is well known that groups I and II introns can propagate as moving elements through different mechanisms: group I by HE activity and group II by reverse protein transcription (Bonen and Vogel, 2001; Hausner, 2003; Lang et al., 2007). Since introns have the ability to disperse throughout the genome, we investigated intron sharing among genes and species. According to our data, there is a high intron sharing rate among fungal species of the order Hypocreales (267 out of 349 introns were shared among species). These findings may be an indicative of horizontal intron transfer. Horizontal intron transfer mechanisms in fungal mitochondrial DNA have already been described in many species, such as *Chrysoporthe* (Kanzi et al., 2016), *Aspergillus* and *Penicillium* (Joardar et al., 2012) and *Armillaria* (Kolesnikova et al., 2019). Moreover, besides the introns sharing in species of the order Hypocreales being more frequent among those taxonomically related, it was also observed for species belonging to different families. Mardanov et al. (2014) identified nine introns in rRNA genes from *Sclerotinia borealis*, but only three introns sequences presented similarity with rRNA introns in the Helotiales order. Other four sequences exhibited similarity with mitochondrial genes from species that are not related to this order. In the same study, it was described that species from this order had the same intron insertion position in the *cox1* gene. In our study, we also checked for the presence of intron sharing by position in mitochondrial genes. We found that the *cox1* gene, followed by the *cob* and *rrnl* genes, had the highest sharing rate among species, displaying respectively 21, 8, and 5 different insertions events. Nevertheless, we also found conserved insertion positions in the species evaluated. Joardar et al. (2012) showed that the *cox1* gene for Aspergillus species presented variable insertions sites. Furthermore, Guha et al. (2018) analyzed 129 fungal species from Ascomycota phylum and found 21 different sites of intron insertion in the gene *cob*. These authors also described that some positions are more common for insertion sites and suggested that these locations may be preferred by HEGs activity. In addition, it was shown that introns insertion site preservation could be related to the host fitness. In *Saccharomyces cervisiae*, for instance, the presence of introns in nuclear and mitochondrial genes helps to resist starvation conditions (Parenteau et al., 2019).

Two IGI sequences classified as type IA located in the *rrnl* gene were the most shared among the fungal species in our analyses and were also detected in the genes *cox1* and *cob.* According to Zubaer et al. (2018), some introns occurring in the *rrnl* gene are well conserved and widespread in many fungal species of the phylum Ascomycota. In Saccharomycetales, the presence of an IGI in the gene *rrnl*, called *omega* intron, has been studied in detail (Goddard and Burt, 1999). The authors observed a cycle of invasion and degeneration for this intron, which, in order to be maintained in mitogenome continue transposing into other genes or genomes (horizontal transfer). This invasion cycle occurs through the presence of HEGs that promote intron insertion into a mitochondrial DNA region. This mechanism of invasion cycle is mainly responsible for intron diversity in mtDNAs, as well as sequence sharing among different fungal species (Goddard and Burt, 1999; Wu and Hao, 2014). IGIs may also transpose via reverse splicing of RNA. This mechanism is based on the recombination of the intron-containing rRNA molecule that is reverse transcribed into DNA and inserted by recombination processes in other sites of the mitochondrial genome (Roman and Woodson, 1995; Hausner, 2003). This mechanism can corroborate that both introns IA detected in the gene *rrnl* are the most shared among species and present in other genes other than *rrnl*. Furthermore, some introns were found exclusively in one species (such as for *Metarhizium anisopliae*) or in a unique gene (*atp8* and *atp9*), not being shared with no other species or gene.

Mitochondrial genes have numerous sequences of HEGs, which are capable of self-mobilization. Since HEGs can interrupt or structurally modify mitochondrial genes (Joardar et al., 2012; Kolesnikova et al., 2019), we evaluated the duplication of mitochondrial genes to the nuclear genome. Duplication was found a common phenomenon among the mitochondrial genes of species belonging to the order Hypocreales, since among the 17 core genes, only *atp8*, *atp9*, and *cox3* were not detected in the nuclear genome. Duplication of NUMTs is a common phenomenon in other kingdoms, such as in mammals (Metazoa) (Tsuji et al., 2012). In Fungi, few studies that have verified duplications of mitochondrial genes in the nuclear genome have been described (Wright and Cummings, 1983; Specht et al., 1992; Brankovics et al., 2018; Wang et al., 2018). The species *Schizopphillum commune* has a duplication of the *atp9* gene (Specht et al., 1992), and in *Podospora anserina* there are mitochondrial plasmids containing *cox1* and *cox3* genes during its senescence (Wright and Cummings, 1983). Furthermore, in *Fusarium gramineraum*, a mitochondrial pangenomic analysis revealed a 3,174 bp NUMT fragment (Brankovics et al., 2018). In our gene ordering analyses, some species, such as *Metacordyceps chlamydosporia*, *Cordyceps militaris* and *Fusarium circinatum* did not show any duplication of mitochondrial genes, as found and described in *Hirsutella thompsonii* (Wang et al., 2018).

Based on the NUMT data, we also evaluated the divergence time by molecular clock analyses using mitochondrial genes that are exclusively from the mitochondrial genome. Molecular clock is used to investigate the timing of phylogenetic events, such as dating the origin of taxonomic groups or events of gene duplication, diversification or gene loss (Weir and Schluter, 2008). Herein, we estimated that the order Hypocreales probably originated around 137.39 Mya. Compared to an estimation based on 638 protein sequences in the nuclear genome, which indicates that Hypocreales originated approximately at 193 Mya (Kubicek et al., 2019). It is well known that the evolutionary rate of fungal mtDNA is close to that of plants, which have the lowest nucleotide substitution rates (Aguileta et al., 2014; Sandor et al., 2018). The evolution rate is based on the percentage of base substitutions in conserved genetic regions. Clark-Walker (1991) demonstrated that the percentage of substitution in mitochondrial genes was lower than that of nuclear genes, suggesting that fungal mitogenomes evolve slower than their respective nuclear genomes. Therefore, the time found in our analyses is expected, since mitochondrial genomes evolve slower than nuclear genomes.

Also, based in the value of $\bar{d\,S}$ of each species, it was possible to estimate the relationship among the genome size and the neutral substitution rates. The more fast evolving a mitogenome is, the larger its size and more abundant in non-coding regions, such as introns, HEGs and uORFs, while the protein coding-region are mostly unchanged. This indicates that the protein-coding genes of mitogenomes are under the influence of negative selection, since the coding regions remains stable independently of the neutral substitutions rates.

Fungal mitochondrial genomes usually contain 14 core protein-coding genes involved in the electron transport chain ATP synthase complex (Kang X. et al., 2017). In addition to these genes, they also have two ribosomal genes and *rps3* protein that are also conserved. In our analysis, most of the species presented the 14 core genes widely described for fungal mitogenomes, the two ribosomal genes and *rps3* protein.

The ordering of the mitochondrial genes of the evaluated species of the order Hypocreales is remarkably conserved. Nonetheless, the species *Acremonium fuci*, *A. chrysogenum* and *Clonostachys rosea* presented the gene *cox2* displaced, while *Nectria cinnabarina* and *Epichloe typhina* displayed a rearrangement for the *nad4l* gene. Differences in genome ordering can be attributed due to non-homologous recombination processes, plasmid sequence integration or presence of transposable elements, such as IGI sequences and HEGs (Gordenin et al., 1993; Lavrov et al., 2002; Rocha, 2003; Repar and Warnecke, 2017).

Species of the *Beauveria* genera exhibited an additional copy of the gene *rps3* as a freestanding gene. The *rps3* gene is the only ribosomal protein encoded by fungal mtDNA and may be located as a freestanding gene or inserted within an intron of the *rrnl* gene (Sethuraman et al., 2009). Gene duplication may occur due to truncated sequences or loss of function. In the mitogenomes of *Beauveria* evaluated, *rps3* copies were almost the same size. Nevertheless, only freestanding copies had coding potential, suggesting that the *rps3* copy located in the *rrnl* gene could be a pseudogene.

In the current study we provided the complete sequencing of the mitogenome of the fungus *Trichoderma harzianum*, a commercially important fungal strain. Comparative analyses of 35 species of the order Hypocreales revealed a structural dynamic in the mitochondrial genome of this well-studied and diverse order of the Fungi kingdom. The genome size variability found was mainly correlated with the presence of non-coding elements, such as introns and HEGs, which could be considered the determinant elements for the shape of mitochondrial genomes, as described in other Orders. Some studies, such as the one described by Mardanov et al. (2014) verified the difference in mitogenomes size and presence of introns in the orders Peltigetales and Helotiales and showed a dynamic of acquisition and loss of introns during evolution. In our work, we estimated that mitogenomes that evolve faster, have longer length of mitogenome and non-coding region.

## Data Availability Statement

The in-house scripts generated for this study can be found in the Supplementary Text S1 and are available in GitHub repository (https://github.com/paulaluize/mitogenomes). The original contributions presented in the study are publicly available. This data can be found here: https://www.ncbi.nlm.nih.gov/nucleotide/; accession number MT263519.

## Author Contributions

PF, BB, VA, EA, and AG-N conceived and designed experiments. PF, RD-P, DA, DB, EA, FB, L-ED-B, and AG-N analyzed the data. PF, FB, L-ED-B, EA, and AG-N wrote the manuscript. All authors read and approved the final manuscript.

## Conflict of Interest

The authors declare that the research was conducted in the absence of any commercial or financial relationships that could be construed as a potential conflict of interest.

## Footnotes

1 http://www.bioinformatics.babraham.ac.uk/projects/fastqc/

2 https://sourceforge.net/projects/bbmap/

3 http://mitos2.bioinf.uni-leipzig.de/index.py

4 http://megasun.bch.umontreal.ca/RNAweasel/

5 https://www.geneious.com

6 www.myleus.com

7 https://www.ncbi.nlm.nih.gov/genome/organelle/

8 https://www.hiv.lanl.gov/content/sequence/SNAP/SNAP.html

## Supplementary Material

FIGURE S1 Pearson’s correlation of genomic features and size of mitochondrial genome. The presence and size of homing endonucleases, introns, genome size, uORFs, coding region, repeats, tRNAs and rRNAs were evaluated. The correlation ranges from −1 (red) to 1 (blue). Asterisks indicate the significance of the correlation, where ^∗^ indicate *p* < 0.05, ^∗∗^ indicate *p* < 0.005, and ^∗∗∗^ indicate *p* < 0.0005.

FIGURE S2 Analysis of size variation of the mitochondrial genes. Green represents the size of the exon that presents the conserved domain that characterizes the gene. Yellow represents the sum of all exons of the gene (aa).

FIGURE S3 Pearson’s correlation between the sizes of introns and homing endonucleases.

FIGURE S4 Phylogenetic analyses of introns classified as subgroup IA. The tree was constructed using Maximum Likelihood with 1000 replicates of bootstrap. The species and gene which the intron was originated are shown for each sequence.

FIGURE S5 Identification of events of mitochondrial gene transfer to the nuclear genomes (NUMT). In this analyses, 18 species with nuclear genome available in public databases were evaluated.

TABLE S1 Accession numbers of mitochondrial and nuclear genomes analyzed in this study.

TABLE S2 Features annotated in the mitogenome of *Trichoderma harzianum* isolate HB324.

TABLE S3 Genomic characteristics of the mitochondrial genomes from Hypocreales order. The table contains information about genome length, features, GC content and repeats.

TABLE S4 Overview of intron sharing analysis based on gene position for the species evaluated in this study.

TEXT S1 Scripts used in this work.

TEXT S2 Mitochondrial genome sequence of *Trichoderma harzianum* HB324.
