# Supplementary figures and images for: Exploring the Relationship Among Divergence Time and Coding and Non-coding Elements in the Shaping of Fungal Mitochondrial Genomes
Source: Front Microbiol. 2020 Apr 29;11:765. doi: 10.3389/fmicb.2020.00765 (PMC7202290; doi:10.3389/fmicb.2020.00765)

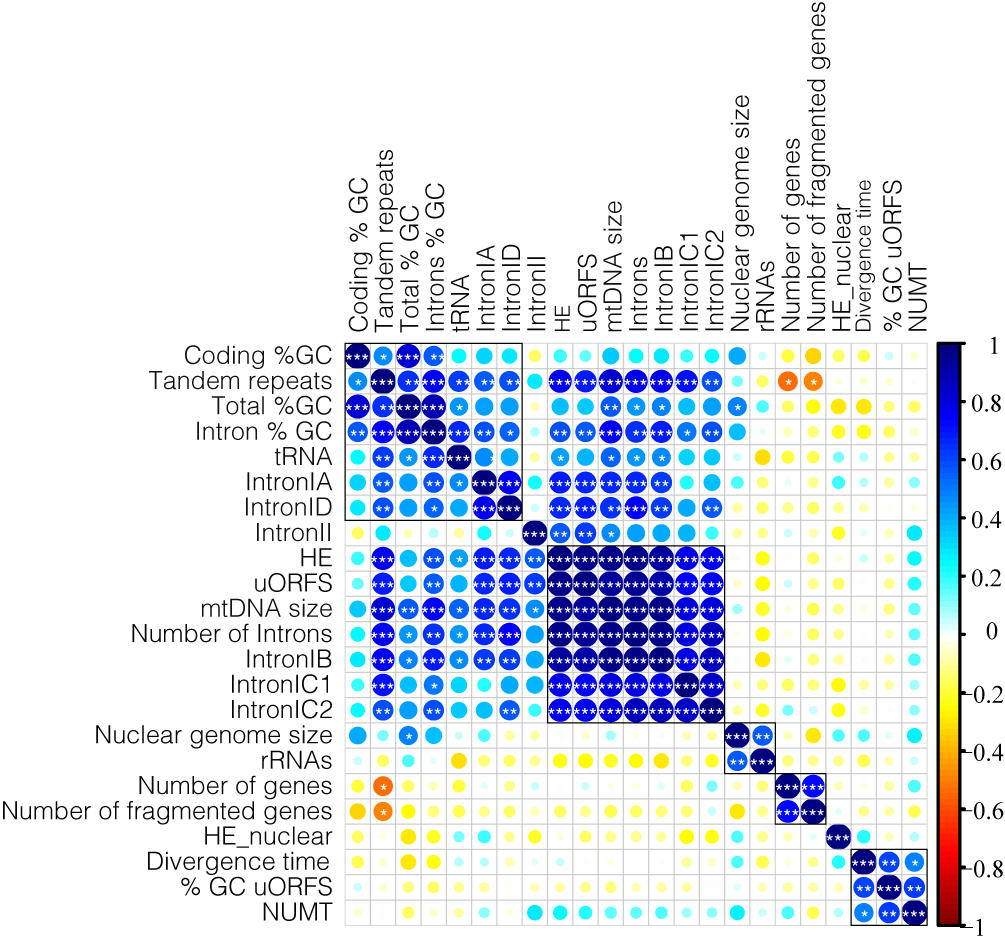

Supplement: FIGURE S1 — Pearson’s correlation of genomic features and size of mitochondrial genome. The presence and size of homing endonucleases, introns, genome size, uORFs, coding region, repeats, tRNAs and rRNAs were evaluated. The correlation ranges from −1 (red) to 1 (blue). Asterisks indicate the significance of the correlation, where ∗ indicate p < 0.05, ∗∗ indicate p < 0.005, and ∗∗∗ indicate p < 0.0005. [file Image_1.TIF]

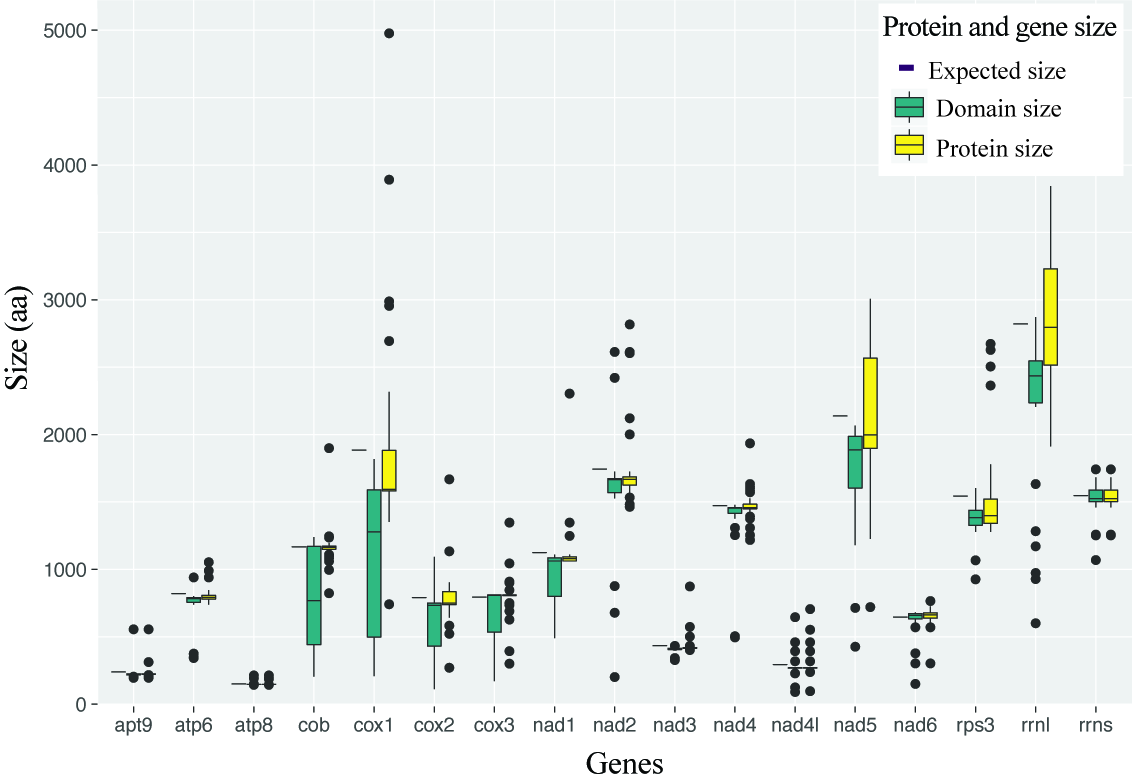

Supplement: FIGURE S2 — Analysis of size variation of the mitochondrial genes. Green represents the size of the exon that presents the conserved domain that characterizes the gene. Yellow represents the sum of all exons of the gene (aa). [file Image_2.TIF]

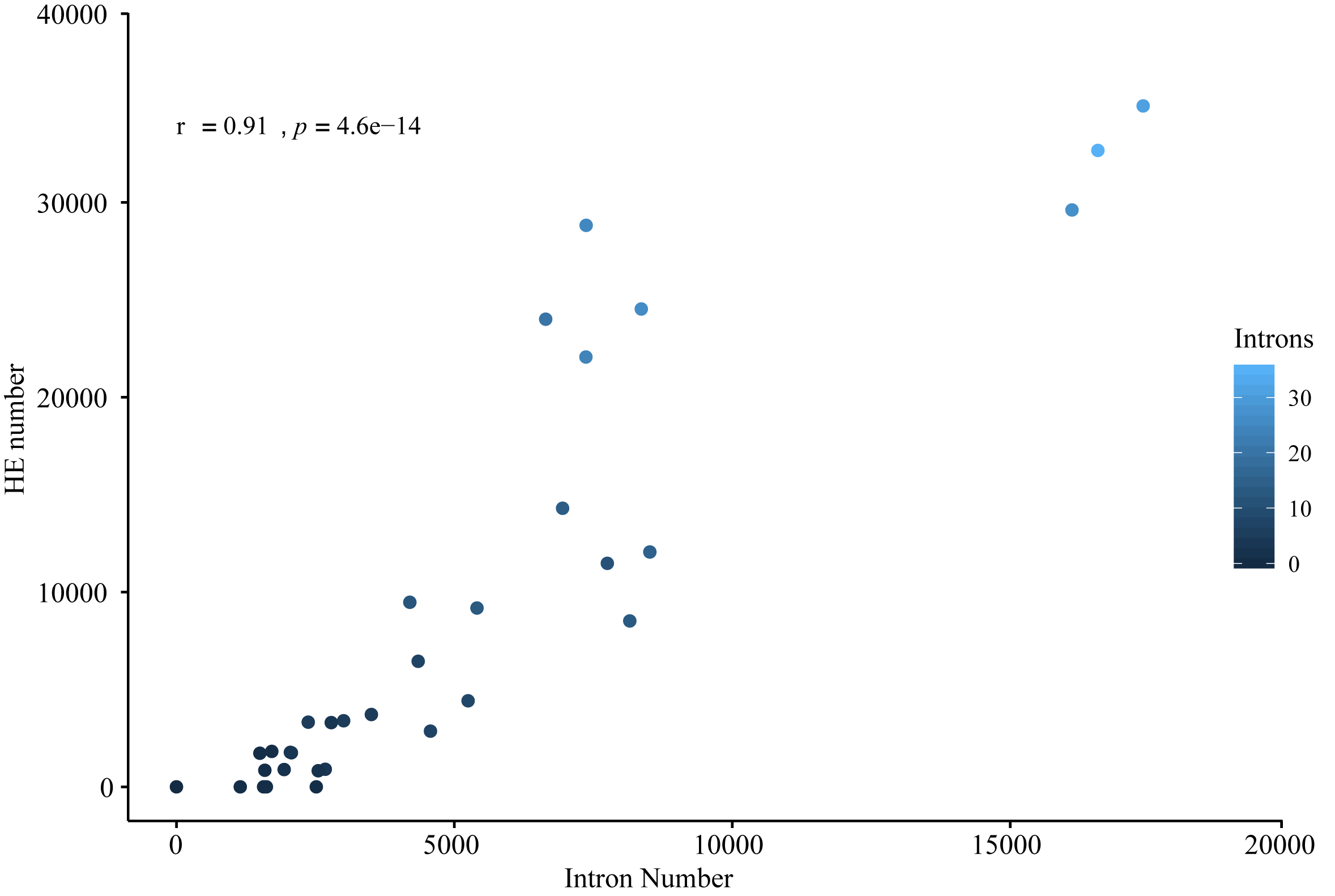

Supplement: FIGURE S3 — Pearson’s correlation between the sizes of introns and homing endonucleases. [file Image_3.TIF]

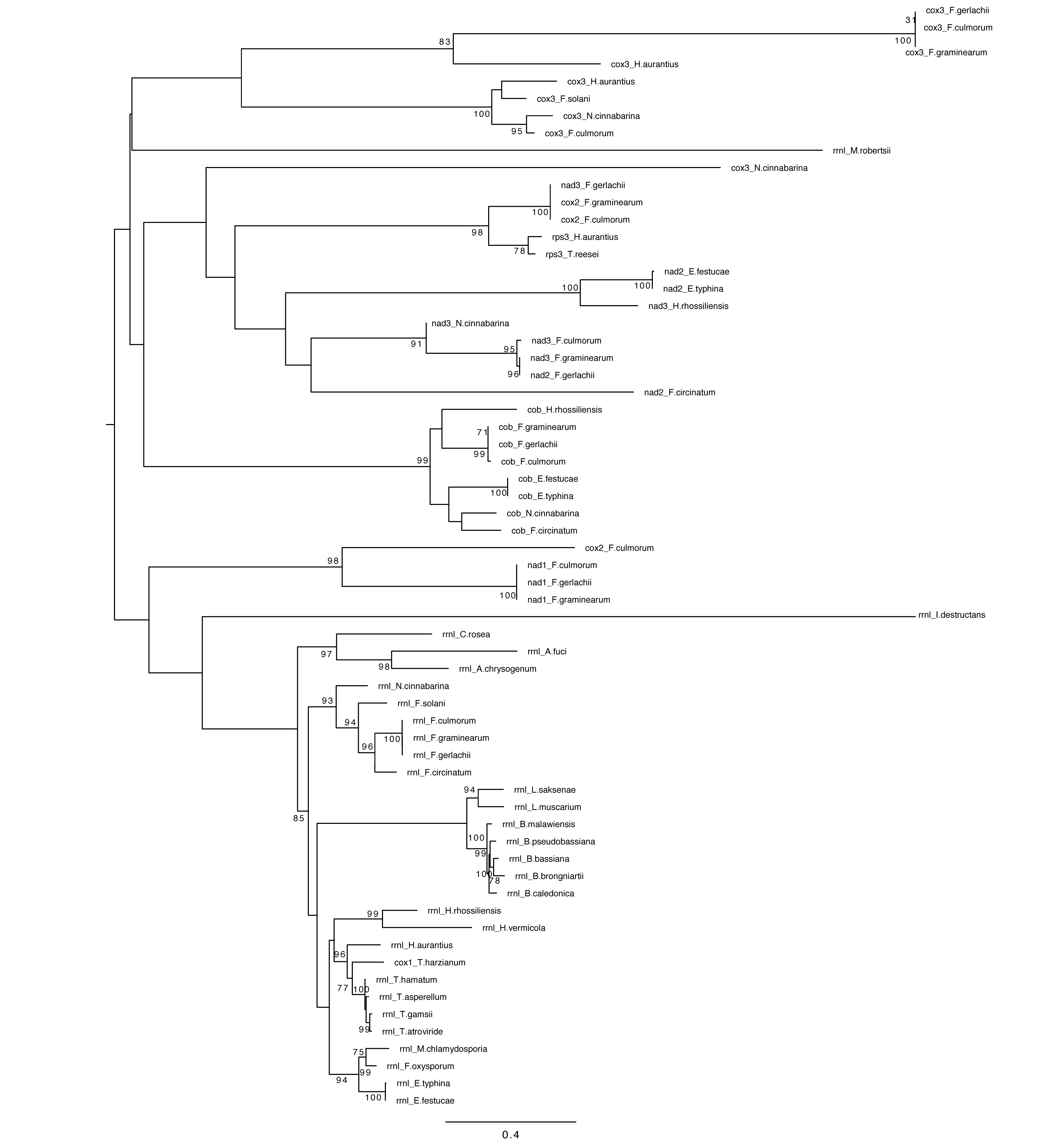

Supplement: FIGURE S4 — Phylogenetic analyses of introns classified as subgroup IA. The tree was constructed using Maximum Likelihood with 1000 replicates of bootstrap. The species and gene which the intron was originated are shown for each sequence. [file Image_4.TIF]

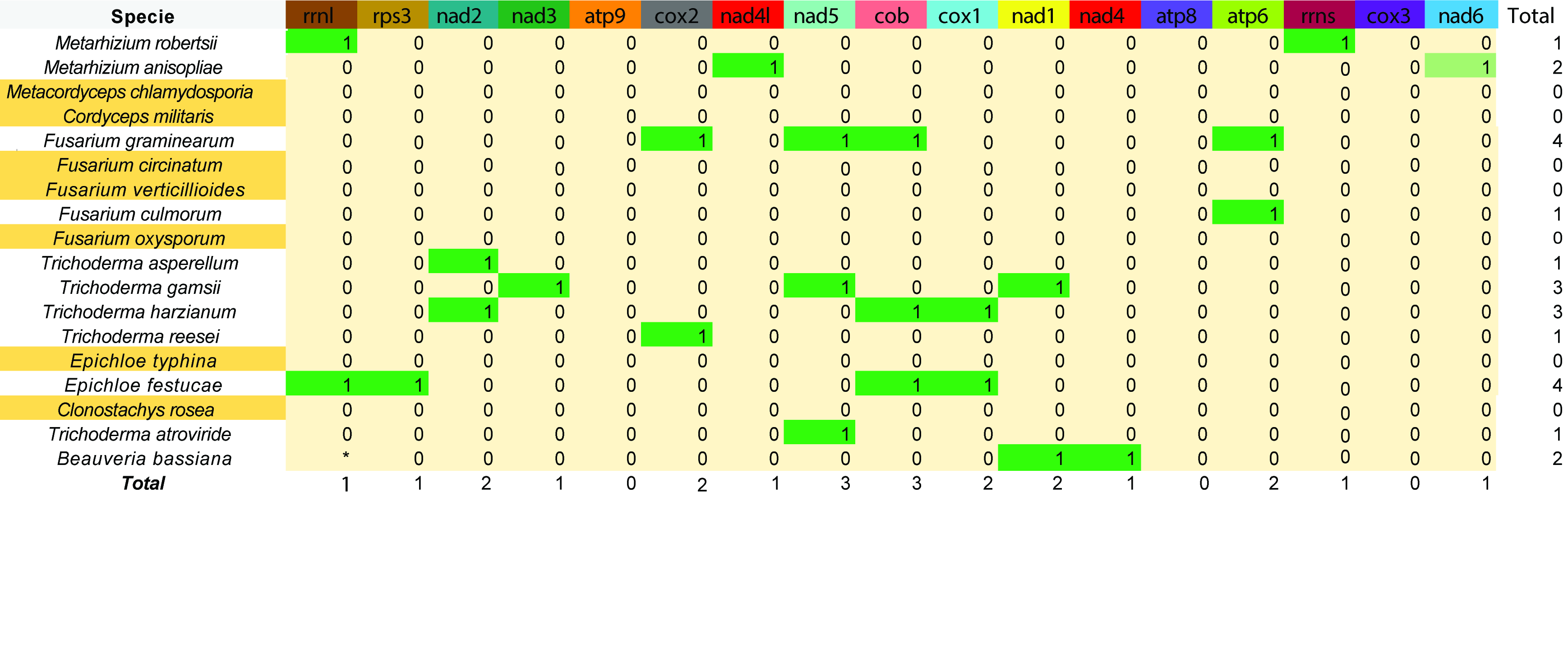

Supplement: FIGURE S5 — Identification of events of mitochondrial gene transfer to the nuclear genomes (NUMT). In this analyses, 18 species with nuclear genome available in public databases were evaluated. [file Image_5.TIF]
