# Supplementary material for: Exploring the Relationship Among Divergence Time and Coding and Non-coding Elements in the Shaping of Fungal Mitochondrial Genomes
Source: Front Microbiol. 2020 Apr 29;11:765. doi: 10.3389/fmicb.2020.00765 (PMC7202290; doi:10.3389/fmicb.2020.00765)
Supplement: TABLE S1 — Accession numbers of mitochondrial and nuclear genomes analyzed in this study. [file Table_1.docx]

**Supplementary Table 1.** Accession number of the mitocondrial and nuclear genomes used in this study.

| **Species** | **Mitochondrial access number** | **Nuclear acess number** | **Reference**  **of mitogenome** |
| --- | --- | --- | --- |
| *Metarhizium robertsii* | JELW01000367 | GCA_000187425 | NA |
| *Metarhizium anisopliae* | NC_008068 | GCA_000814975 | 1 |
| *Metacordyceps chlamydosporia* | NC_022835 | GCA_001653235 | 2 |
| Cordyceps brongniartii | NC_011194 | ------------------------ | 3 |
| *Cordyceps militaris* | NC_022834 | GCA_000225605 | 4 |
| *Acremonium chrysogenum* | KF757229 | ------------------------ | 5 |
| *Acremonium fuci* | NC_029851 | ------------------------ | 6 |
| *Fusarium graminearum* | KP966551 | GCA_000240153 | 7 |
| *Fusarium solani* | NC_016680 | ------------------------ | 8 |
| *Fusarium circinatum* | NC_022681 | GCA_000497325 | 9 |
| *Gibberella moniliformis* | NC_016687 | GCA_000149555 | 8 |
| *Fusarium culmorum* | NC_026993 | GCA_900074845 | 10 |
| *Fusarium gerlachii* | KM486533 | ------------------------ | 11 |
| *Fusarium oxysporum* | AY945289 | GCA_000149955 | 12 |
| *Trichoderma asperellum* | KR952346 | GCA_003025105 | NA |
| *Trichoderma gamsii* | NC_030218 | GCA_001481775 | 13 |
| *Trichoderma atroviride* | JGI1185329 | GCA_000171015 | NA |
| *Trichoderma hamatum* | NC_036144 | ------------------------ | NA |
| *Trichoderma harzianum* HB324 | Supplementary Text S1 | GCA_003025095 | This study |
| *Trichoderma reesei* | NC_003388 | GCA_000167675 | 14 |
| *Lecanicillium muscarium* | NC_004514 | ------------------------ | 15 |
| *Lecanicillium saksenae* | NC_028330 | ------------------------ | 16 |
| *Beauveria bassiana* | NC_010652 | GCA_000280675 | NA |
| Beauveria pseudobassiana | NC_022708 | ------------------------ | 17 |
| *Beauveria malawiensis* | NC_030635 | ------------------------ | NA |
| *Beauveria caledonica* | NC_030636 | ------------------------ | NA |
| *Hirsutella minnesotensis* | NC_027660 | ------------------------ | NA |
| *Hirsutella rhossiliensis* | NC_030164 | ------------------------ | 18 |
| *Hirsutella vermicola* | NC_036610 | ------------------------ | 19 |
| *Hypomyces aurantius* | NC_030206 | ------------------------ | 20 |
| *Nectria cinnabarina* | NC_030252 | ------------------------ | 21 |
| *Ilyonectria destructans* | NC_030340 | ------------------------ | NA |
| *Epichloe typhina* | NC_032063 | GCA_000308955 | NA |
| *Epichloe festucae* | NC_032064 | GCA_003814445 | NA |
| *Clonostachys rosea* | NC_036667 | GCA_000963775 | 22 |

**References**

1. Ghikas, D. V., Kouvelis, V. N., & Typas, M. A. (2006). The complete mitochondrial genome of the entomopathogenic fungus Metarhizium anisopliae var. anisopliae: gene order and trn gene clusters reveal a common evolutionary course for all Sordariomycetes, while intergenic regions show variation. *Archives of microbiology*, *185*(5), 393.
2. Lin, R., Liu, C., Shen, B., Bai, M., Ling, J., Chen, G., ... & Xie, B. (2015). Analysis of the complete mitochondrial genome of Pochonia chlamydosporia suggests a close relationship to the invertebrate-pathogenic fungi in Hypocreales. *BMC microbiology*, *15*(1), 5.
3. Ghikas, D. V., Kouvelis, V. N., & Typas, M. A. (2010). Phylogenetic and biogeographic implications inferred by mitochondrial intergenic region analyses and ITS1-5.8 S-ITS2 of the entomopathogenic fungi Beauveria bassiana and B. brongniartii. *BMC microbiology*, *10*(1), 174.
4. Sung, G. H. (2015). Complete mitochondrial DNA genome of the medicinal mushroom Cordyceps militaris (Ascomycota, Cordycipitaceae). *Mitochondrial DNA*, *26*(5), 789-790.
5. Eldarov, M. A., Mardanov, A. V., Beletsky, A. V., Dumina, M. V., Ravin, N. V., & Skryabin, K. G. (2015). Complete mitochondrial genome of the cephalosporin-producing fungus Acremonium chrysogenum. *Mitochondrial DNA*, *26*(6), 943-944.
6. Konovalova, O., & Logacheva, M. (2016). Mitochondrial genome of two marine fungal species. *Mitochondrial DNA Part A*, *27*(6), 4280-4281
7. Brankovics, B., Kulik, T., Sawicki, J., Bilska, K., Zhang, H., de Hoog, G. S., ... & van Diepeningen, A. D. (2018). First steps towards mitochondrial pan-genomics: detailed analysis of Fusarium graminearum mitogenomes. *PeerJ*, *6*, e5963.
8. Al-Reedy, R. M., Malireddy, R., Dillman, C. B., & Kennell, J. C. (2012). Comparative analysis of Fusarium mitochondrial genomes reveals a highly variable region that encodes an exceptionally large open reading frame. *Fungal Genetics and Biology*, *49*(1), 2-14.
9. Fourie, G., Van der Merwe, N. A., Wingfield, B. D., Bogale, M., Tudzynski, B., Wingfield, M. J., & Steenkamp, E. T. (2013). Evidence for inter-specific recombination among the mitochondrial genomes of Fusarium species in the Gibberella fujikuroi complex. *BMC genomics*, *14*(1), 605.
10. Kulik, T., Brankovics, B., Sawicki, J., & van Diepeningen, A. (2016). The complete mitogenome of Fusarium culmorum. *Mitochondrial DNA Part A*, *27*(4), 2425-2426.
11. Kulik, T., Brankovics, B., Sawicki, J., & van Diepeningen, A. D. (2016). The complete mitogenome of Fusarium gerlachii. *Mitochondrial DNA Part A*, *27*(3), 1895-1896.
12. Pantou, M. P., Kouvelis, V. N., & Typas, M. A. (2008). The complete mitochondrial genome of Fusarium oxysporum: insights into fungal mitochondrial evolution. *Gene*, *419*(1-2), 7-15.
13. Deng, Y., Zhang, Q., Ming, R., Lin, L., Lin, X., Lin, Y., ... & Wen, Z. (2016). Analysis of the mitochondrial genome in Hypomyces aurantius reveals a novel twintron complex in fungi. *International journal of molecular sciences*, *17*(7), 1049.
14. Chambergo, F. S., Bonaccorsi, E. D., Ferreira, A. J., Ramos, A. S., Ferreira, J. R., Abrahao-Neto, J., ... & El-Dorry, H. (2002). Elucidation of the metabolic fate of glucose in the filamentous fungus Trichoderma reesei using expressed sequence tag (EST) analysis and cDNA microarrays. *Journal of Biological Chemistry*, *277*(16), 13983-13988.
15. Kouvelis, V. N., Ghikas, D. V., & Typas, M. A. (2004). The analysis of the complete mitochondrial genome of Lecanicillium muscarium (synonym Verticillium lecanii) suggests a minimum common gene organization in mtDNAs of Sordariomycetes: phylogenetic implications. *Fungal genetics and Biology*, *41*(10), 930-940.
16. Xin, B., Lin, R., Shen, B., Mao, Z., Cheng, X., & Xie, B. (2017). The complete mitochondrial genome of the nematophagous fungus Lecanicillium saksenae. *Mitochondrial DNA Part A*, *28*(1), 52-53.
17. Oh, J., Kong, W. S., & Sung, G. H. (2015). Complete mitochondrial genome of the entomopathogenic fungus Beauveria pseudobassiana (Ascomycota, Cordycipitaceae). *Mitochondrial DNA*, *26*(5), 777-778.
18. Zhang, W., Zhang, X., Li, K., Wang, C., Cai, L., Zhuang, W., ... & Liu, X. (2018). Introgression and gene family contraction drive the evolution of lifestyle and host shifts of hypocrealean fungi. *Mycology*, *9*(3), 176-188.
19. Zhang, Y. J., Zhang, H. Y., Liu, X. Z., & Zhang, S. (2017). Mitochondrial genome of the nematode endoparasitic fungus Hirsutella vermicola reveals a high level of synteny in the family Ophiocordycipitaceae. *Applied microbiology and biotechnology*, *101*(8), 3295-3304.
20. Deng, Y., Zhang, Q., Ming, R., Lin, L., Lin, X., Lin, Y., ... & Wen, Z. (2016). Analysis of the mitochondrial genome in Hypomyces aurantius reveals a novel twintron complex in fungi. *International journal of molecular sciences*, *17*(7), 1049.
21. Wang, X. C., Zeng, Z. Q., & Zhuang, W. Y. (2016). The complete mitochondrial genome of the important phytopathogen Nectria cinnabarina (Hypocreales, Ascomycota). *Mitochondrial DNA Part A*, *27*(6), 4670-4671.
22. Wang, X. C., Zeng, Z. Q., & Zhuang, W. Y. (2017). The complete mitochondrial genome of the important mycoparasite Clonostachys rosea (Hypocreales, Ascomycota). *Mitochondrial DNA Part B*, *2*(1), 180-181.
