# Supplementary material for: Exploring the Relationship Among Divergence Time and Coding and Non-coding Elements in the Shaping of Fungal Mitochondrial Genomes
Source: Front Microbiol. 2020 Apr 29;11:765. doi: 10.3389/fmicb.2020.00765 (PMC7202290; doi:10.3389/fmicb.2020.00765)
Supplement: TABLE S2 — Features annotated in the mitogenome of Trichoderma harzianum isolate HB324. [file Table_2.docx]

**Supplementary Table S2.** Features annotated in the mitogenome of Trichoderma harzianum HB324.

| Feature | Start | End | Size | Codon |
| --- | --- | --- | --- | --- |
| *rrnL*-a | 31 | 1771 | 1741 |  |
| Thar.rrnlP1633 | 1634 | 2528 | 895 |  |
| lagli | 1768 | 2100 | 333 | TTT/AAA |
| Thar.rps3P3416 | 3417 | 4983 | 901 |  |
| *rps3* | 4030 | 5084 | 1437 | ATG/TAA |
| orf167 | 4581 | 5084 | 504 | CTT/AAT |
| orf295 | 5156 | 6094 | 888 | CTT/AAT |
| lagli_1 | 641 | 1504 | 864 | CTT/AAT |
| *rrnL*_b | 5890 | 6763 | 601 |  |
| trnT(tgt) | 6792 | 6862 | 71 |  |
| trnE(ttc) | 6868 | 6940 | 73 |  |
| trnM_2(cat) | 6941 | 7011 | 71 |  |
| trnM_1(cat) | 7159 | 7231 | 73 |  |
| trnL2(taa) | 7234 | 7316 | 83 |  |
| trnA(tgc) | 7408 | 7479 | 72 |  |
| trnF_0(gaa) | 7485 | 7557 | 73 |  |
| trnK(ttt) | 7650 | 7722 | 73 |  |
| trnL1(tag) | 7795 | 7877 | 83 |  |
| trnQ_0(ttg) | 7955 | 8027 | 73 |  |
| trnQ_1(ttg) | 8120 | 8186 | 67 |  |
| trnQ_2(ttg) | 8279 | 8342 | 64 |  |
| trnH(gtg) | 8359 | 8431 | 73 |  |
| trnM_0(cat) | 8529 | 8599 | 71 |  |
| *nad2* | 8685 | 10352 | 1668 | ATG/TAA |
| *nad3* | 10353 | 10766 | 405 | ATG/GAA |
| *atp9* | 10957 | 11181 | 219 | ATG/GTT |
| orf447 | 11352 | 12698 | 1344 | ATG/GTT |
| *cox2*_a | 12734 | 12818 | 84 | ATG/CAA |
| Thar.cox2P124 | 11479 | 11710 | 232 |  |
| lagli_0 | 11709 | 12698 | 891 | ATG/AAT |
| *cox2*_b | 12817 | 13399 | 657 | ATG/AAT |
| trnR(acg) | 13484 | 13554 | 71 |  |
| *nad4l* | 13888 | 14157 | 267 | ATG/AAA |
| *nad5* | 14157 | 15485 | 1329 | ATG/ATA |
| *cob*_a | 16541 | 16936 | 396 | ATG/TAC |
| giy | 16913 | 17818 | 717 | ATG/AAT |
| Thar.cobP1388 | 17929 | 18051 | 123 |  |
| *cob*_b | 18140 | 18900 | 759 | ATG/AAT |
| trnC(gca) | 19029 | 19097 | 69 |  |
| *cox1*-a | 19593 | 19805 | 213 | ATG/GTC |
| orf324 | 19806 | 20780 | 975 | ATG/GTG |
| giy | 19800 | 20780 | 840 | ATG/TAA |
| Thar.cox1P1231 | 20824 | 22174 | 1350 |  |
| *cox1*-b | 21057 | 22430 | 1371 | ATG/AGT |
| trnX(tct) | 22528 | 22598 | 71 |  |
| *nad1* | 22984 | 24045 | 1089 | ATG/TAA |
| *nad4*_a | 24223 | 25680 | 1419 | ATG/TAA |
| trnF_1(---) | 25764 | 25835 | 72 |  |
| *nad4*_b | 25867 | 26085 | 216 | ATG/TAA |
| *atp8* | 26169 | 2315 | 147 | ATG/ATA |
| *atp6* | 26434 | 27216 | 783 | ATG/TAA |
| giy | 27296 | 27376 | 81 | ATG/TAA |
| *rrnS* | 27796 | 29310 | 1514 |  |
| trnY(gta) | 29348 | 29432 | 85 |  |
| trnD(gtc) | 29526 | 29599 | 74 |  |
| trnS1(gct) | 29605 | 29688 | 84 |  |
| trnN(gtt) | 29693 | 29764 | 72 |  |
| *cox3* | 29810 | 30304 | 495 | ATG/TCA |
| trnG(tcc) | 30682 | 30752 | 71 |  |
| *nad6* | 30863 | 31580 | 645 | ATG/TAA |
| trnV(tac) | 31531 | 31603 | 73 |  |
| trnI(gat) | 31753 | 31824 | 72 |  |
| trnS2(tga) | 31849 | 31933 | 85 |  |
| trnW(tca) | 31940 | 32012 | 73 |  |
| trnP(tgg) | 32050 | 32121 | 72 |  |

The Feature column indicates the putative gene identified in the mitogenome of *Trichoderma harzianum*. The Start and End columns represents the start and end position of each feature annotated on *T. harzianum* genome. The Size column, indicates the size of each Feature and the Codon column, indicates the first and last codon of each gene.
