# Supplementary material for: Exploring the Relationship Among Divergence Time and Coding and Non-coding Elements in the Shaping of Fungal Mitochondrial Genomes
Source: Front Microbiol. 2020 Apr 29;11:765. doi: 10.3389/fmicb.2020.00765 (PMC7202290; doi:10.3389/fmicb.2020.00765)
Supplement: TABLE S3 — Genomic characteristics of the mitochondrial genomes from Hypocreales order. The table contains information about genome length, features, GC content and repeats. [file Table_3.docx]

**Supplementary Table 3**. Genomic characteristics of mitogenomes from fungal species analyzed in this study.

| **Specie** | **Genome size (nt)** | **Number of genes** | **Coding region (nt)** | **% GC** | **Number of tRNA** | **Number of rRNAs** | **Number of introns** | **Number of HE** | **Number of uORFS** | **Number of Repeats** |
| --- | --- | --- | --- | --- | --- | --- | --- | --- | --- | --- |
| *Metarhizium robertsii* | 24,944 | 38 | 20,937 | 28.27 | 24 | 2 | 1 | 0 | 1 | 15 |
| *Metarhizium anisopliae* | 24,673 | 41 | 21,205 | 28.39 | 23 | 2 | 0 | 0 | 1 | 14 |
| *Metacordyceps chlamydosporia* | 25,615 | 39 | 20,924 | 28.28 | 25 | 2 | 1 | 0 | 4 | 19 |
| *Beauveria* brongniartii | 33,926 | 42 | 28,198 | 27.34 | 25 | 2 | 5 | 4 | 8 | 15 |
| *Cordyceps militaris* | 33,277 | 43 | 29,416 | 26.79 | 27 | 2 | 8 | 3 | 7 | 21 |
| Acremonium chrysogenum | 27,266 | 45 | 20,383 | 26.54 | 25 | 2 | 3 | 1 | 5 | 21 |
| *Acremonium fuci* | 24,565 | 45 | 20,383 | 28.77 | 28 | 2 | 1 | 1 | 1 | 17 |
| *Fusarium graminearum* | 97,364 | 86 | 14,644 | 31.72 | 27 | 2 | 34 | 46 | 43 | 33 |
| *Fusarium solani* | 62,978 | 57 | 54,570 | 28.9 | 25 | 2 | 15 | 22 | 25 | 41 |
| *Fusarium circinatum* | 67,109 | 44 | 28,267 | 31.44 | 26 | 2 | 15 | 16 | 19 | 30 |
| *Fusarium verticillioides* | 53,753 | 50 | 36,572 | 32.60 | 27 | 2 | 4 | 6 | 6 | 25 |
| *Fusarium culmorum* | 103,844 | 90 | 49,869 | 31.7 | 27 | 2 | 39 | 41 | 32 | 29 |
| *Fusarium gerlachii* | 93,428 | 83 | 45,059 | 31.90 | 27 | 2 | 34 | 34 | 37 | 31 |
| *Fusarium oxysporum* | 34,477 | 17 | 19,029 | 31 | 25 | 2 | 2 | 0 | 2 | 19 |
| *Trichoderma asperellum* | 29,999 | 43 | 19,564 | 27.78 | 26 | 2 | 3 | 2 | 3 | 19 |
| *Trichoderma atroviride* | 30,481 | 42 | 18,699 | 28.10 | 25 | 2 | 2 | 1 | 4 | 20 |
| *Trichoderma gamsii* | 29,303 | 47 | 20,468 | 28.25 | 26 | 2 | 1 | 0 | 3 | 22 |
| *Trichoderma hamatum* | 32,763 | 48 | 23,451 | 27.67 | 25 | 2 | 5 | 4 | 5 | 21 |
| *Trichoderma harzianum* | 32,277 | 43 | 16,879 | 27.74 | 25 | 2 | 5 | 5 | 5 | 23 |
| *Trichoderma reesei* | 42,130 | 44 | 26,481 | 27.24 | 25 | 2 | 11 | 10 | 11 | 28 |
| *Lecanicillium muscarium* | 24,499 | 42 | 21,137 | 27.15 | 22 | 2 | 1 | 0 | 1 | 12 |
| *Lecanicillium saksenae* | 25,919 | 43 | 20,978 | 26.53 | 26 | 2 | 1 | 0 | 4 | 21 |
| *Beauveria bassiana* | 29,961 | 42 | 23,836 | 27.25 | 25 | 2 | 3 | 2 | 6 | 25 |
| Beauveria pseudobassiana | 28,006 | 43 | 22,656 | 27.54 | 25 | 2 | 2 | 1 | 3 | 23 |
| *Beauveria malawiensis* | 44,135 | 56 | 35,044 | 26.72 | 25 | 2 | 13 | 14 | 15 | 24 |
| *Beauveria caledonica* | 38,316 | 54 | 28,556 | 26.26 | 26 | 2 | 7 | 8 | 13 | 31 |
| *Hirsutella minnesotensis* | 52,245 | 56 | 35,913 | 28.42 | 25 | 2 | 12 | 15 | 16 | 28 |
| *Hirsutella rhossiliensis* | 62,483 | 47 | 32,463 | 28.21 | 27 | 2 | 13 | 27 | 17 | 39 |
| *Hirsutella vermicola* | 53,793 | 54 | 32,936 | 25.27 | 25 | 2 | 7 | 1 | 11 | 53 |
| *Hypomyces aurantius* | 71,638 | 71 | 49,360 | 28.31 | 25 | 2 | 20 | 44 | 28 | 35 |
| *Nectria cinnabarina* | 69,895 | 42 | 21,053 | 28.70 | 25 | 2 | 26 | 39 | 25 | 35 |
| *Ilyonectria destructans* | 42,895 | 46 | 21,856 | 28.23 | 27 | 2 | 1 | 2 | 14 | 27 |
| *Epichloe typhina* | 84,630 | 81 | 47,098 | 26.96 | 25 | 2 | 24 | 50 | 42 | 51 |
| *Epichloe festucae* | 88,744 | 85 | 50,976 | 27.52 | 25 | 2 | 25 | 44 | 46 | 53 |
| *Clonostachys rosea* | 40,921 | 42 | 28,507 | 27.9 | 25 | 2 | 8 | 7 | 10 | 26 |
