# Supplementary material for: Exploring the Relationship Among Divergence Time and Coding and Non-coding Elements in the Shaping of Fungal Mitochondrial Genomes
Source: Front Microbiol. 2020 Apr 29;11:765. doi: 10.3389/fmicb.2020.00765 (PMC7202290; doi:10.3389/fmicb.2020.00765)
Supplement: Supplementary file 10 [file Table_5.DOCX]

#This script is part of supplementary documents of "Impact of Introns and Homing Endonucleases on Structural Mitogenome Shaping in Hypocreales"

#submitted to Frontiers in Microbiology, section Fungi and Their Interactions

#Manuscript ID: 531057

#Authors: Paula Fonseca, Fernanda Badotti, Ruth De-Paula, Daniel Araújo, Dener Eduardo Bortolini, Luiz-Eduardo Del-Bem, Vasco Ariston De Carvalho Azevedo,

#Bertram Brenig, Eric Roberto Guimarães Rocha Aguiar, Aristóteles Góes-Neto

#This script uses the NCBI API to make a query and retrieve data from GenBank with all gene annotation of target species

#Using a text file as input, the script can retrieve data from multiples species at time

#The result is a '.cds' file, with name, ID, size of genome, start, end positions and the name of all genes

#******************************************************************************#

# Run the code in Python 3+ #

#******************************************************************************#

# -*- Coding: UTF-8 -*-

#coding: utf-8

import sys

import urllib.request

import re

import os.path

from os import path

#This function reads the target(s) id(s) specie(s) from a 'txt' file

def readIDs(fileName_txt):

ids_file=open(fileName_txt,'r')

query_IDs = []

for line in ids_file:

#the '[accn]' substring is added as requirement by NCBI API

query_IDs.append(line.rstrip()+"[accn]")

ids_file.close()

return query_IDs

#This function will try to retrieve the data of a target specie using NCBI API. The result is a xml string with all data.

def getXMLNCBI(str_ID):

urlBase = "https://eutils.ncbi.nlm.nih.gov/entrez/eutils/"

#create URL for esearch

url = urlBase+"esearch.fcgi?db=nuccore&term="+str_ID+"&usehistory=y"

f = urllib.request.urlopen(url)

#Read xml from url

xml = f.read()

strXml=xml.decode("utf-8")

#If WebEnv e QueryKey exists in this firstxml, fetch the URL query

objRe = re.search('<WebEnv>(\S+)<\/WebEnv>',strXml)

webEnv = objRe.group()

webEnv = webEnv[8:len(webEnv)-9]

objRe = re.search('<QueryKey>(\d+)<\/QueryKey>',strXml)

queryKey = objRe.group()

queryKey = queryKey[10:len(queryKey)-11]

#URL efetch

url = urlBase+"efetch.fcgi?db=nuccore&query_key="+queryKey+"&WebEnv="+webEnv+"&rettype=gb&retmode=xml"

f = urllib.request.urlopen(url)

xml_esearch_bin = f.read()

xml_esearch = xml_esearch_bin.decode("utf-8")

return xml_esearch

#This function generates xml and cds files with the data retrieved from NCBI. The CDS file will contain all the annotated genes within the genome of a specie

def generateXMLCDS(item, xml_esearch, key_words):

genome_ID=""

genome_size=0

genome_size_CDS=0

#To treat gene overlapping, the array cds_vector will store the nucleotides positons that are part of a coding region

#If a nucleotide is part of CDS, then its position on cds_vector will be '1', while the positions with '0' will represent the nucleotides of non coding region

cds_vector=[]

#The index_key_words is a counter that controls the exploration of the ordered indendation and alignment in the xml format, retrieving the gene data when found

index_key_words=0

#Open output files .xml and .cds

output_xml_file=open(item[:len(item)-6]+".xml",'w')

output_cds_file=open(item[:len(item)-6]+".cds","w")

for line in xml_esearch:

output_xml_file.write(line+"\n")

#The command below search for the key_word indicated by index_key_words in the present line of the xml

id_str_found=line.find(key_words[index_key_words])

if(id_str_found!=-1):

#if it is the first index_key_word, get the genome ID and set to next key_word

if (index_key_words==0):

genome_ID=line[id_str_found+len(key_words[index_key_words]):line.find("<",id_str_found+1)]

index_key_words=index_key_words+1

#If it is the second one, get genome total size and set to next key_word

elif (index_key_words==1):

genome_size=int(line[id_str_found+len(key_words[index_key_words]):line.find("<",id_str_found+1)])

#Instantiate cds_vector with size + 1 of the whole genome. The 0 position will not be used

cds_vector = [0]*(genome_size+1)

genome_size_CDS=0

index_key_words=index_key_words+1

#In the third key_word, get specie name header, write in screen and cds file and set to next key_word

elif (index_key_words==2):

output_cds_file.write(line[id_str_found+len(key_words[index_key_words]):line.find("<",id_str_found+1)]+"\n")

output_cds_file.write("Genome ID: "+genome_ID+"\n")

output_cds_file.write("Genome size: "+str(genome_size)+"\n")

output_cds_file.write("Genes:\n")

print(line[id_str_found+len(key_words[index_key_words]):line.find("<",id_str_found+1)])

print("Genome ID: "+genome_ID)

print("Genome size: "+str(genome_size))

index_key_words=index_key_words+1

#Here we get the information if the next data is part of a gene and go further into the xml with another key_word

elif (index_key_words==3):

index_key_words=index_key_words+1

#In the fifth one, we get the string with the start and end positions of the gene, calling a function that print the values on screen and cds file,

#besides marking the nuclotides positions of the gene on cds_vector

elif (index_key_words==4):

#Treat a Join if necessary

if (line.find("join")==-1):

rangeCDS=re.sub('[^0-9.]','',line)

write_start_end_gene(rangeCDS,output_cds_file,cds_vector)

else:

output_cds_file.write("\n\n")

rangeCDS=re.sub('[^0-9.,]','',line)

rangeCDS1=rangeCDS[:rangeCDS.find(",")]

write_start_end_gene(rangeCDS1,output_cds_file,cds_vector)

print()

rangeCDS2=rangeCDS[rangeCDS.find(",")+1:]

write_start_end_gene(rangeCDS2,output_cds_file,cds_vector)

index_key_words=index_key_words+1

#We go further into the xml indendation

elif (index_key_words==5):

index_key_words=index_key_words+1

#And get the gene name, printing on screen and cds file

elif (index_key_words==6):

gene_name=line[line.find("<GBQualifier_value>")+19:line.find("</GBQualifier_value>",18)]

output_cds_file.write("#"+gene_name+"\n")

print(" ("+str(gene_name)+")")

#Then we retrocede 3 positions in index_key_value to look for another genes

index_key_words=index_key_words-3

#Get the number of nucleotides in coding regions

genome_size_CDS=sum(cds_vector)

print("Sum of nucleotides in the coding regions (CDS) of the genome ID= "+genome_ID+": "+str(genome_size_CDS)+" of "+str(genome_size)+" nucleotides ("+str(round(genome_size_CDS*100/genome_size,2))+"%)")

output_cds_file.write("Sum of nucleotides in the coding regions (CDS) of the genome: "+str(genome_size_CDS)+" of "+str(genome_size)+" nucleotides ("+str(round(genome_size_CDS*100/genome_size,2))+"%)")

output_cds_file.close()

output_xml_file.close()

#This function extracts from a string the start and end position of a gene, print the data on screen and on cds file and register the position of all nucleotides on cds_vector

#by changing the '0' value to 1. The change only occurs once for a nucleotide.

def write_start_end_gene(range_gene, output_cds_file,cds_vector):

indexRange=range_gene.find("..")

print("\t\t"+range_gene[:indexRange]+"\t"+range_gene[indexRange+2:], end = '')

output_cds_file.write(range_gene[:indexRange]+";"+range_gene[indexRange+2:])

for i in range(int(range_gene[:indexRange]),int(range_gene[indexRange+2:])+1):

if (cds_vector[i]==0):

cds_vector[i]=cds_vector[i]+1

#Function to check if files are OK

def checkInputFiles():

#Check if all the necessary files names are passed as arguments

if (len(sys.argv)!=2 or sys.argv[1].find(".txt")==-1):

print ("\nUsage:\npython getCDSGenBank.py [file_path_name.txt]")

sys.exit(0)

fileName_txt=sys.argv[1]

#Check if path/files exists

if (not (path.exists(fileName_txt))):

print("\nOne or more files not found! Check the path and file names.\n")

exit(0)

return fileName_txt

def main():

fileName_txt=checkInputFiles()

query_IDs=readIDs(fileName_txt)

#The key words are used to read the '.xml' format return by NCBI API and extract the genes data from it

key_words=["<GBSeq_locus>", "<GBSeq_length>", "<GBSeq_definition>", "<GBFeature_key>gene</GBFeature_key>", "<GBFeature_location>","<GBQualifier_name>","<GBQualifier_value>"]

#This variable store ids that returns a empty result

error_ids=""

for item in query_IDs:

print("\n\nQueryng ID: "+item[:len(item)-6]+"\n\n")

#Get xml with GenBAnk data from NCBI

xml_esearch=getXMLNCBI(item)

if (xml_esearch.find("<ERROR>Empty result - nothing to do</ERROR>")==-1):

xml_esearch=xml_esearch.splitlines()

generateXMLCDS(item,xml_esearch,key_words)

else:

error_ids=error_ids+item[:len(item)-6]+"\n"

print("\n--------------------------------------------------------------------------------------------\n")

#If some ID returned empty, list them

if (len(error_ids)>0):

print("\n--------------------------------------------------------------------------------------------\n")

print("\nThe following IDs returned a empty result:\n"+error_ids+"\nCheck these IDs and try again\n")

print("\n--------------------------------------------------------------------------------------------\n")

if __name__ == '__main__':

main()

#This script is part of supplementary documents of "Impact of Introns and Homing Endonucleases on Structural Mitogenome Shaping in Hypocreales"

#submitted to Frontiers in Microbiology, section Fungi and Their Interactions

#Manuscript ID: 531057

#Authors: Paula Fonseca, Fernanda Badotti, Ruth De-Paula, Daniel Araújo, Dener Eduardo Bortolini, Luiz-Eduardo Del-Bem, Vasco Ariston De Carvalho Azevedo,

#Bertram Brenig, Eric Roberto Guimarães Rocha Aguiar, Aristóteles Góes-Neto

#This script uses a gff and fasta files of a target species to get the sequences of genes of interest (GOI)

#The gff file provide the start and end positions of each GOI.

#The output file is in fasta format, with the names (preceded by '>') and the sequence of genes of interest (GOI)

#******************************************************************************#

# Run the code in Python 3+ #

#******************************************************************************#

import sys

import os.path

from os import path

def checkInputFiles():

#Check if all the necessary files names are passed as arguments

if (len(sys.argv)!=3 or sys.argv[1].find(".gff")==-1 or sys.argv[2].find(".fasta")==-1):

print ("\nUsage:\npython getGeneSeqGff.py [file_path_name.gff] [file_path_name.fasta]\n\n")

sys.exit(0)

gff_file_name=sys.argv[1]

fasta_file_name=sys.argv[2]

#Check if path/files exists

if (not (path.exists(gff_file_name) or path.exists(fasta_file_name))):

print("\nOne or more files not found! Check the path and file names.\n")

exit(0)

return gff_file_name, fasta_file_name

#Reads whole sequence from input fasta file

def readFasta(fasta_file):

#Position 0 of whole_genome will not be used

whole_genome=" "

for line in fasta_file:

if (line.find(">")==-1):

whole_genome=whole_genome+line.strip()

fasta_file.close()

return whole_genome

#This function check if the gene_name is present in the whitelist array, case true, it saves the name and sequence of the gene

def search_genes(gene_name,gene_sequence, output_file, GOI):

for GOI in GOI:

if (gene_name.startswith(GOI)):

output_file.write(">"+gene_name+"\n"+gene_sequence+"\n\n")

break

#Read the gff file to extract data.

#The gff file contains 1 gene per row with several values ordered by 'tab'. Its straight forward to get the name and positions of a single gene

#and retrieve th sequence from the whole_genome

def readGffSelGenes(GOI, gff_file, output_file, whole_genome):

for line in gff_file:

values=line.split("\t")

#Start position is at index 3

start_gene_position=values[3]

#End position is at index 4

end_gene_position=values[4]

#Name is at index 8

gene_name=values[8][values[8].find("Name=")+5:].strip()

#Get gene sequence

gene_sequence=whole_genome[int(start_gene_position):int(end_gene_position)+1]

#Verify if it is on gene_whitelist to save in output file

search_genes(gene_name,gene_sequence,output_file,GOI)

def main():

GOI={"rrnL","rps3","nad2","nad3","atp9","cox2","nad4l","nad5","cob","cox1","nad1","nad4","atp8","atp6","rrnS","cox3","nad6"}

gff_file_name, fasta_file_name=checkInputFiles()

gff_file=open(gff_file_name,'r')

fasta_file=open(fasta_file_name,'r')

whole_genome=readFasta(fasta_file)

#Get ID specie from gff file name

#The strip will remove '.\' that appear on console in Windows 10 before path\filename

if (os.name=="nt"):

gff_file_name=gff_file_name.strip(".\\")

output_file_name=gff_file_name[0:gff_file_name.find(".")]+"_GOI.fasta"

#Open output file with '_GOI.fasta' extension

output_file=open(output_file_name,'w')

readGffSelGenes(GOI,gff_file,output_file, whole_genome)

gff_file.close()

output_file.close()

print("\n\n____________________________________________________________")

print("\nResults saved in: "+output_file_name)

print("____________________________________________________________\n\n\n")

if __name__ == '__main__':

main()

#This script is part of supplementary documents of "Impact of Introns and Homing Endonucleases on Structural Mitogenome Shaping in Hypocreales"

#submitted to Frontiers in Microbiology, section Fungi and Their Interactions

#Manuscript ID: 531057

#Authors: Paula Fonseca, Fernanda Badotti, Ruth De-Paula, Daniel Araújo, Dener Eduardo Bortolini, Luiz-Eduardo Del-Bem, Vasco Ariston De Carvalho Azevedo,

#Bertram Brenig, Eric Roberto Guimarães Rocha Aguiar, Aristóteles Góes-Neto

#This script uses the output file of Mfannot to list and save uORFs of a target species

#As described in https://github.com/BFL-lab/Mfannot, Mfannot is a program for annotation of mitochondrial and plastid genomes

#******************************************************************************#

# Run the code in Python 3+ #

#******************************************************************************#

# -*- Coding: UTF-8 -*-

#coding: utf-8

import sys

import os.path

from os import path

#Function to check if files are OK

def checkMfannotFile():

#Check if all the necessary files names are passed as arguments

if (len(sys.argv)!=2):

print ("\nUsage:\npython getuORFs.py [file_path_name]")

sys.exit(0)

mfannot_file_name=sys.argv[1]

#Check if path/files exists

if (not (path.exists(mfannot_file_name))):

print("\nOne or more files not found! Check the path and file names.\n")

exit(0)

#Open input file

input_file=open(mfannot_file_name,'r')

#Check if input file is a Mfannot

if (input_file.readline().find("mfannot")==-1):

print("\nThe file is empty or is not a mfannot output file\n")

input_file.close()

exit(0)

#Get ID specie from Mfannot file name

#The strip will remove '.\' that appear on console in Windows 10 before path\filename

if (os.name=="nt"):

mfannot_file_name=mfannot_file_name.strip(".\\")

output_file_name=mfannot_file_name[0:mfannot_file_name.find(".")]

print(mfannot_file_name)

print(output_file_name)

#Open uORFs output file

output_file=open(output_file_name+".uORFs",'w')

return input_file, output_file

#This function look if a string contain a uORfs_name and store it in uORfs_name_vector

def findOrfs(str_name, uORfs_name_vector):

if (str_name.find("orf")!=-1):

uORfs_name_vector.append(str_name)

def getuORFSNamesMfannot(uORfs_name_vector, input_file):

#find_str_gene is a boolean variable that signalize where the block of gene names start and end

#The Mfannot file lists all gene names in a tab format starting at line 4

find_str_gene=0

#Loop to read the Mfannot input file

for line in input_file:

#If the string "List of genes added", then the boolean find_str_gene receives 1, signalizing that we are reading the block with gene names

if (line.find("List of genes added")!=-1):

find_str_gene=1

#If find_str_gene is true, then we read gene names

if (find_str_gene==1):

#Check if it is at end of gene names block

if (line.find("end mfannot")!=-1):

find_str_gene=0 #para terminar de ler até o end do mfannot.

#The gene names are structured in 3 columns of regular spaced sizes, so we read each one and stores in uORfs_name_vector

else:

findOrfs(line[8:29].rstrip(' '),uORfs_name_vector)

findOrfs(line[29:50].rstrip(' '),uORfs_name_vector)

findOrfs(line[50:70].rstrip(' '),uORfs_name_vector)

def getuORFsStartEndSeq(uORfs_name_vector, input_file,output_file):

for uORfs_name in uORfs_name_vector:

#This sets the position of reading the input_file at the start

input_file.seek(0)

#This boolean tell us when a sequence of a specific uORF begins

bool_start_seq=0

#orf_seq store the orf's sequence

orf_seq=""

orf_start_position=""

orf_end_position=""

detailed_orf_name=""

#num_index store the index +1 after the number position in sequences lines

num_index=-1

#Loop to read the input_file

for line in input_file:

#When we find the start line with the uORFS_name, bool_start_seq receives 1 (true)

if (line.find("-" + uORfs_name)!=-1 and line.find(" ==> start")!=-1):

detailed_orf_name=line[1:line.find(" ==> start")].strip()

bool_start_seq=1

else:

if (bool_start_seq==1):

#num_index store the index +1 after the number position in sequences lines

num_index=line.find(" ",2)

#If orf_start_position is empty and bool_start_seq==1, then we get the start position of the orf

if (orf_start_position==""):

orf_start_position=line[:num_index].strip()

#Check if its the end of the sequence of uORfs_name, then break case true

if (line.find("-" + uORfs_name)!=-1 and line.find(" ==> end")!=-1):

print(">"+detailed_orf_name)

print("+"+orf_start_position)

print("-"+str(orf_end_position))

print("@"+orf_seq)

output_file.write(">"+detailed_orf_name+"\n")

output_file.write("+"+orf_start_position+"\n")

output_file.write("-"+str(orf_end_position)+"\n")

output_file.write("@"+orf_seq+"\n\n")

#Here we reset variables and let the loop go to the end, as is possible to have another copy

#forward in the file

orf_seq=""

orf_start_position=""

orf_end_position=""

detailed_orf_name=""

bool_start_seq=0

elif(line.find(";")==-1):

orf_seq=orf_seq + line[num_index:].strip()

#Calculate the orf_end_position

orf_end_position=int(line[:num_index].strip())+len(line[num_index:].strip())-1

def main():

input_file,output_file=checkMfannotFile()

#uORfs_name_vector is a array that stores the name of the uORFs listed in Mfannot file

uORfs_name_vector=[]

getuORFSNamesMfannot(uORfs_name_vector, input_file)

getuORFsStartEndSeq(uORfs_name_vector, input_file, output_file)

output_file.close()

input_file.close()

if __name__ == '__main__':

main()

#This script is part of supplementary documents of "Impact of Introns and Homing Endonucleases on Structural Mitogenome Shaping in Hypocreales"

#submitted to Frontiers in Microbiology, section Fungi and Their Interactions

#Manuscript ID: 531057

#Authors: Paula Fonseca, Fernanda Badotti, Ruth De-Paula, Daniel Araújo, Dener Eduardo Bortolini, Luiz-Eduardo Del-Bem, Vasco Ariston De Carvalho Azevedo,

#Bertram Brenig, Eric Roberto Guimarães Rocha Aguiar, Aristóteles Góes-Neto

#This script calculates the GC content of whole genome, CDS and genes in the uORFs file

#The files used are as follow:

# -uORFs - Generated by getuORFs.py script

# -cds - Generated by getGenesGenBank.py script

# -fasta - Donwloaded from NCBI

#******************************************************************************#

# Run the code in Python 3+ #

#******************************************************************************#

# -*- Coding: UTF-8 -*-

#coding: utf-8

import sys

import re

import os.path

from os import path

def checkInputFiles():

# #Check if all the necessary files names are passed as arguments

if (len(sys.argv)!=4 or sys.argv[1].find(".uORFs")==-1 or sys.argv[2].find(".cds")==-1 or sys.argv[3].find(".fasta")==-1):

print ("\nUsage:\npython GC_Contet_uORFs.py [file_path_name.uORFs] [file_path_name.cds] [file_path_name.fasta]\n")

sys.exit(0)

#Get path/file names

uORFs_file_name=sys.argv[1]

cds_file_name=sys.argv[2]

fasta_file_name=sys.argv[3]

#Check if path/files exists

if (not (path.exists(uORFs_file_name) or path.exists(cds_file_name) or path.exists(fasta_file_name))):

print("\nOne or more files not found! Check the path and file names.\n")

exit(0)

#Open input files

uORFs_file=open(uORFs_file_name,'r')

cds_file=open(cds_file_name, 'r')

fasta_file=open(fasta_file_name,'r')

#Open output files. The ID filename in uORFs file is used to generate the result files ('.gct' and '.csv')

if (os.name=="nt"):

uORFs_file_name=uORFs_file_name.strip(".\\")

output_file_name=uORFs_file_name[0:uORFs_file_name.find(".")]

output_gct_file=open(output_file_name+".gct",'w')

#The csv file was generated to help analyze the results. Each row of 'csv' file represent a nucleotide position in the whole genome.

#The idea is as follows:

#Row value= 0 = indicates the nucleotide belongs a non coding region

#Row value= 1 = indicates the nucleotide belongs a coding region

#Row value= 2 = indicates the nucleotide belongs a coding region and to 2 genes.

#Row value= 10 = indicates the nucleotide belongs a non coding region and to a uORF

#Row value= 11 = indicates the nucleotide belongs a coding region and to a uORF

#Row value= 12 = indicates the nucleotide belongs a coding region, to a uORF and 2 genes

#Row value= 22 = indicates the nucleotide belongs a coding region, to 2 uORFs and 2 genes

#and so on

#The strip will remove '.\' that appear on console in Windows 10 before path\filename

output_csv_file=open(output_file_name+".csv",'w')

return uORFs_file,cds_file,fasta_file,output_gct_file,output_csv_file

#Based on cds file, this function creates a numerical array (genome_array) that represents where the coding, non coding and uORFs are, returning it

def createGenomeArray(cds_file):

#genome_array represent the whole genome. Position 0 is not used.

genome_array=[]

genome_size=0

#Loop to get data from cds file

for line in cds_file:

#get total genome size from cds file

if (line.rfind("Genome size: ")!=-1):

genome_size=int(line[13:])

#Instantiate size of genome in genome_array and populates with value=0

genome_array=[0]*(genome_size+1)

#Get start and end positions of coding regions (genes on cds)

if (line.find(";")!=-1):

aux_index=line.find(";")

line=line.strip()

start=int(line[:aux_index])

end=int(line[aux_index+1:line.find("#")])

#Loop to register nucleotides that belong to coding regions, based on start and end positions retrieved

#This adds +1 every time a nucleotide belong to a gene

for i in range(start,end+1):

genome_array[i]=genome_array[i]+1

return genome_array

def checkCG(nc_char):

if (nc_char=='C' or nc_char=='G'):

return True

else:

return False

#This function calculates the GC content of the coding and non coding regions of a sequence. Using the genome_array as input,

#its possible to determinte the GC content in coding and non coding region. As well check results in csv file about them and the instersection

# with the uORFs

def gcContentCalc(start, end, sequence, genome_array):

#seq_cds store the sequence of nucleotides that are part of the coding region. Those nucleotides that are not part of the coding region are replaced by '-'

seq_cds=""

#sum_gc_nuc store the sum of GC nucleotides in the sequence

sum_gc_nuc=0

#sum_gc_nuc_cds store the sum of GC nucleotides that are part of coding region in the sequence

sum_gc_nuc_cds=0

#nuc_cds store the sum of ALL nucleotides that are part of coding region in the sequence

nuc_cds=0

#For every nucleotide in the sequence

for i in range(start,end+1):

#Check if its part of a coding region in genome_array

#Because uORFs nucleotides adds +10 to genome_array and is possible that they are not part of coding regions,

#we get the remainder of division by 10

if (genome_array[i]%10>0):

#if it is G or C

if (checkCG(sequence[i-start])):

#Add 1 to sum sum_gc_nuc_cds

sum_gc_nuc_cds=sum_gc_nuc_cds+1

#Add nucleotide to seq_cds

seq_cds=seq_cds+sequence[i-start]

#And 1 to nuc_cds

nuc_cds=nuc_cds+1

#if not part of coding region, '-' replace the nucleotide in seq_cds

else:

seq_cds=seq_cds+'-'

#Indenpedent of being part of coding region

#If C or G

if (checkCG(sequence[i-start])):

#Add 1 to sum_gc_nuc

sum_gc_nuc=sum_gc_nuc+1

#Adding +10 to genome_array will help later check where are the nucleotides that belong to uORFs in csv file

#Values greater than or equal 10

genome_array[i]=genome_array[i]+10

#GC_nc_ratio_cds show the proportion of GC nucleotides in the coding region of the sequence

GC_nc_ratio_cds=0

if (nuc_cds>0):

GC_nc_ratio_cds=sum_gc_nuc_cds/nuc_cds*100

#The next command line returns: proportion of GC nucleotides in the sequence

#Nucleotides in coding region of the sequence

#Total of GC nucleotides in the sequence

#Total of GC nucleotides in the coding region of the sequence

#Total of nucleotides in the coding region of the sequence

#Size of sequence

#and the proportion of GC nucleotides in the coding region of the sequence

return sum_gc_nuc/(end+1-start)*100, seq_cds, sum_gc_nuc, sum_gc_nuc_cds,nuc_cds,end+1-start, GC_nc_ratio_cds

#This function read the whole genome from fasta file

def readWholeGenome(fasta_file):

#The position 0 of whole_genome will not be used

whole_genome=" "

for line in fasta_file:

if(line[0]!=">"):

line=line.upper()

whole_genome=whole_genome+line.strip()

fasta_file.close()

return whole_genome

#Function that calculate uORFs GC content in coding and non coding regions

def uORFsFileGCCalc(uORFs_file, genome_array, output_gct_file):

name_orf=""

#Total of GC nucleotides in ORFs

gc_total_orfs=0

#Total of GC nucleotides in ORFs that are part of coding regions

gc_total_orfs_cds=0

#Total size in nucleotides of the ORFs

sum_size_uorfs=0

#Total size in nucleotides of the ORFs in coding regions

sum_size_uorfs_cds=0

for line in uORFs_file:

if (line.find(">")!=-1):

name_orf=line[1:]

elif (line.find("+")!=-1):

start_orf=int(line[1:])

elif (line.find("-")!=-1):

end_orf=int(line[1:])

elif (line.find("@")!=-1):

seq_orf=line[1:].upper()

print("____________________________________________________________")

print(name_orf)

#call function that calculate GC Content and update genome_array

ratio_GC_orf,seq_in_cds, total_GC_nc_orf, total_GC_nc_orf_cds, total_nc_orf_cds,size_orf,ratio_GC_orf_cds=gcContentCalc(start_orf, end_orf, seq_orf,genome_array)

print("uORf original sequence:\n"+seq_orf+"\nuORF sequence in CDS:\n"+seq_in_cds)

print(start_orf, end_orf)

print("GC Content of Orf:",round(ratio_GC_orf,2))

print("GC Content of Orf in CDS:",round(ratio_GC_orf_cds,2))

output_gct_file.write(name_orf)

output_gct_file.write(str(start_orf)+","+str(end_orf)+"\n")

output_gct_file.write("uORf original sequence:\n"+seq_orf.rstrip()+"\nuORF sequence in CDS:\n"+seq_in_cds+"\n")

output_gct_file.write("Conteudo GC Orf: "+str(round(ratio_GC_orf,2))+"\nConteudo GC Orf CDS: "+str(round(ratio_GC_orf_cds,2))+"\n\n")

gc_total_orfs=gc_total_orfs + total_GC_nc_orf

gc_total_orfs_cds=gc_total_orfs_cds + total_GC_nc_orf_cds

sum_size_uorfs=sum_size_uorfs+size_orf

sum_size_uorfs_cds=sum_size_uorfs_cds+total_nc_orf_cds

return gc_total_orfs,gc_total_orfs_cds,sum_size_uorfs,sum_size_uorfs_cds

#Calculate GC content in whole Genome

def wholeGenomeGCCalc(output_csv_file,output_gct_file,whole_genome, genome_array, gc_total_orfs, gc_total_orfs_cds, sum_size_uorfs, sum_size_uorfs_cds):

#Total of nucleotides in the whole genome that belongs to coding regions

sum_nc_genome_cds=0

#Total of nucleotides in the whole genome that belongs to non coding regions

sum_nc_genome_noncod=0

#Total of GC nucleotides in the whole genome that belongs to coding regions

sum_GC_nc_cds=0

#Total of GC nucleotides in the whole genome that belongs to coding regions

sum_GC_nc_noncod=0

genome_size=len(genome_array)-1

for i in range(1,len(genome_array)):

output_csv_file.write(str(genome_array[i])+"\n")

#Check if nucleotide is part of coding region

#Because uORFs nucleotides adds +10 to genome_array and is possible that they are not part of coding regions,

#we get the remainder of division by 10

if (genome_array[i]%10>0):

if (checkCG(whole_genome[i])):

sum_GC_nc_cds= sum_GC_nc_cds+1

sum_nc_genome_cds=sum_nc_genome_cds+1

else:

if (checkCG(whole_genome[i])):

sum_GC_nc_noncod= sum_GC_nc_noncod+1

sum_nc_genome_noncod=sum_nc_genome_noncod+1

print("____________________________________________________________")

print("\n")

print("------------------------------------------------------------------------------------------------------------------------------------")

print("Whole genome total size = "+str(genome_size)+" nucleotides, where "+ str(sum_nc_genome_cds)+" nucleotides ("+str(round(sum_nc_genome_cds/genome_size*100,2)) \

+"%) belongs to coding regions (CDS) and "+ str(sum_nc_genome_noncod)+" nucleotides ("+str(round(sum_nc_genome_noncod/genome_size*100,2))+"%) belongs to non coding regions (NC)")

print("Whole genome GC content = "+str(sum_GC_nc_cds+sum_GC_nc_noncod)+" of "+str(genome_size)+" nucleotides ("+str(round((sum_GC_nc_cds+sum_GC_nc_noncod)/genome_size*100,2))+"%)")

print("GC content in coding regions = "+str(sum_GC_nc_cds)+" of "+str(sum_nc_genome_cds)+" nucleotides ("+str(round(sum_GC_nc_cds/sum_nc_genome_cds*100,2))+"%)")

print("GC content in non coding regions = "+str(sum_GC_nc_noncod)+" of "+str(sum_nc_genome_noncod)+" nucleotides (" +str(round(sum_GC_nc_noncod/sum_nc_genome_noncod*100,2))+"%)")

print("uORFs total size = "+ str(sum_size_uorfs) + " nucleotides, corresponding to " + str(round(sum_size_uorfs/genome_size*100,2))+ "% "+"of whole genome")

print("uORfs GC content = " +str(gc_total_orfs)+" of "+str(sum_size_uorfs)+" nucleotides ("+str(round(gc_total_orfs/sum_size_uorfs*100,2))+"%)")

print("uORFs total size in coding regions (CDS) = "+ str(sum_size_uorfs_cds) + " nucleotides")

print("uORFs total size in non coding regions (NC) = "+ str(sum_size_uorfs-sum_size_uorfs_cds) + " nucleotides")

print("uORFs GC content in coding regions (CDS) = " +str(gc_total_orfs_cds)+" of "+str(sum_size_uorfs_cds) + " nucleotides ("+ str(round(gc_total_orfs_cds/sum_size_uorfs_cds*100,2))+"%)")

if (sum_size_uorfs-sum_size_uorfs_cds!=0):

print("uORFs GC content in non coding regions (NC) = " +str(gc_total_orfs-gc_total_orfs_cds)+" of "+str(sum_size_uorfs-sum_size_uorfs_cds) + " nucleotides ("+ \

str(round((gc_total_orfs-gc_total_orfs_cds)/(sum_size_uorfs-sum_size_uorfs_cds)*100,2))+"%)")

print("------------------------------------------------------------------------------------------------------------------------------------")

output_gct_file.write("\n")

output_gct_file.write("------------------------------------------------------------------------------------------------------------------------------------\n")

output_gct_file.write("Whole genome total size = "+str(genome_size)+" nucleotides, where "+ str(sum_nc_genome_cds)+" nucleotides ("+str(round(sum_nc_genome_cds/genome_size*100,2)) \

+"%) belongs to coding regions (CDS) and "+ str(sum_nc_genome_noncod)+" nucleotides ("+str(round(sum_nc_genome_noncod/genome_size*100,2))+"%) belongs to non coding regions (NC)\n")

output_gct_file.write("Whole genome GC content = "+str(sum_GC_nc_cds+sum_GC_nc_noncod)+" of "+str(genome_size)+" nucleotides ("+str(round((sum_GC_nc_cds+sum_GC_nc_noncod)/genome_size*100,2))+"%)\n")

output_gct_file.write("GC content in coding regions = "+str(sum_GC_nc_cds)+" of "+str(sum_nc_genome_cds)+" nucleotides ("+str(round(sum_GC_nc_cds/sum_nc_genome_cds*100,2))+"%)\n")

output_gct_file.write("GC content in non coding regions = "+str(sum_GC_nc_noncod)+" of "+str(sum_nc_genome_noncod)+" nucleotides (" +str(round(sum_GC_nc_noncod/sum_nc_genome_noncod*100,2))+"%)\n")

output_gct_file.write("uORFs total size = "+ str(sum_size_uorfs) + " nucleotides, corresponding to " + str(round(sum_size_uorfs/genome_size*100,2))+ "% "+"of whole genome\n")

output_gct_file.write("uORfs GC content = " +str(gc_total_orfs)+" of "+str(sum_size_uorfs)+" nucleotides ("+str(round(gc_total_orfs/sum_size_uorfs*100,2))+"%)\n")

output_gct_file.write("uORFs total size in coding regions (CDS) = "+ str(sum_size_uorfs_cds) + " nucleotides\n")

output_gct_file.write("uORFs total size in non coding regions (NC) = "+ str(sum_size_uorfs-sum_size_uorfs_cds) + " nucleotides\n")

output_gct_file.write("uORFs GC content in coding regions (CDS) = " +str(gc_total_orfs_cds)+" of "+str(sum_size_uorfs_cds) + " nucleotides ("+ str(round(gc_total_orfs_cds/sum_size_uorfs_cds*100,2))+"%)\n")

if (sum_size_uorfs-sum_size_uorfs_cds!=0):

output_gct_file.write("uORFs GC content in non coding regions (NC) = " +str(gc_total_orfs-gc_total_orfs_cds)+" of "+str(sum_size_uorfs-sum_size_uorfs_cds) + " nucleotides ("+ \

str(round((gc_total_orfs-gc_total_orfs_cds)/(sum_size_uorfs-sum_size_uorfs_cds)*100,2))+"%)\n")

output_gct_file.write("------------------------------------------------------------------------------------------------------------------------------------\n")

def main():

uORFs_file,cds_file,fasta_file,output_gct_file,output_csv_file =checkInputFiles()

#Call function that reads data from 'cds' file, creating genome_array

genome_array = createGenomeArray(cds_file)

#Call function to read whole genome from fasta file

whole_genome=readWholeGenome(fasta_file)

#Call function to calculate GC Content of uORfs

gc_total_orfs, gc_total_orfs_cds, sum_size_uorfs, sum_size_uorfs_cds=uORFsFileGCCalc(uORFs_file,genome_array,output_gct_file)

#Call function to calculate GC Content of whole genome

wholeGenomeGCCalc(output_csv_file,output_gct_file, whole_genome, genome_array, gc_total_orfs, gc_total_orfs_cds, sum_size_uorfs, sum_size_uorfs_cds)

print("\n\nResults saved on: "+str(output_gct_file.name)+" e "+str(output_csv_file.name)+"\n")

uORFs_file.close()

cds_file.close()

output_csv_file.close()

output_gct_file.close()

if __name__ == '__main__':

main()
